# MYC-mediated resistance to trametinib and HCQ in PDAC is overcome by CDK4/6 and lysosomal inhibition

**DOI:** 10.1084/jem.20221524

**Published:** 2023-01-31

**Authors:** Mark R. Silvis, Dilru Silva, Riley Rohweder, Sophia Schuman, Swapna Gudipaty, Amanda Truong, Jeffrey Yap, Kajsa Affolter, Martin McMahon, Conan Kinsey

**Affiliations:** 1 Huntsman Cancer Institute, University of Utah, Salt Lake City, UT, USA; 2 Department of Oncological Sciences, University of Utah, Salt Lake City, UT, USA; 3 ARUP Laboratories, Salt Lake City, UT, USA; 4 Intermountain Medical Center, Murray, UT, USA; 5 Department of Radiology, University of Utah, Salt Lake City, UT, USA; 6 Department of Pathology, University of Utah, Salt Lake City, UT, USA; 7 Department of Dermatology, University of Utah, Salt Lake City, UT, USA; 8 Department of Internal Medicine, Division of Oncology, University of Utah, Salt Lake City, UT, USA

## Abstract

Pharmacological inhibition of KRAS>RAF>MEK1/2>ERK1/2 signaling has provided no clinical benefit to patients with pancreatic ductal adenocarcinoma (PDAC). Interestingly, combined inhibition of MEK1/2 (with trametinib [T]) plus autophagy (with chloroquine [CQ] or hydroxychloroquine [HCQ]) demonstrated striking anti-tumor effects in preclinical models and in a patient (Patient 1). However, not all patients respond to the T/HCQ regimen, and Patient 1 eventually developed resistant disease. Here we report that primary or acquired resistance is associated with focal DNA copy number gains encompassing *c-MYC*. Furthermore, ectopic expression of c-MYC in PDAC cell lines rendered them T/HCQ resistant. Interestingly, a CDK4/6 inhibitor, palbociclib (P), also induced autophagy and overrode c-MYC–mediated T/HCQ resistance, such that P/HCQ promoted regression of T/HCQ-resistant PDAC tumors with elevated c-MYC expression. Finally, P/HCQ treatment of Patient 1 resulted in a biochemical disease response. These data suggest that elevated c-MYC expression is both a marker and a mediator of T/HCQ resistance, which may be overcome by the use of P/HCQ.

## Teaser

Inhibition of CDK4/6 and lysosome function overcomes c-MYC–mediated cell cycle entry in the face of trametinib and chloroquine co-treatment in pancreatic cancer.

## Graphical Abstract

**Figure:**
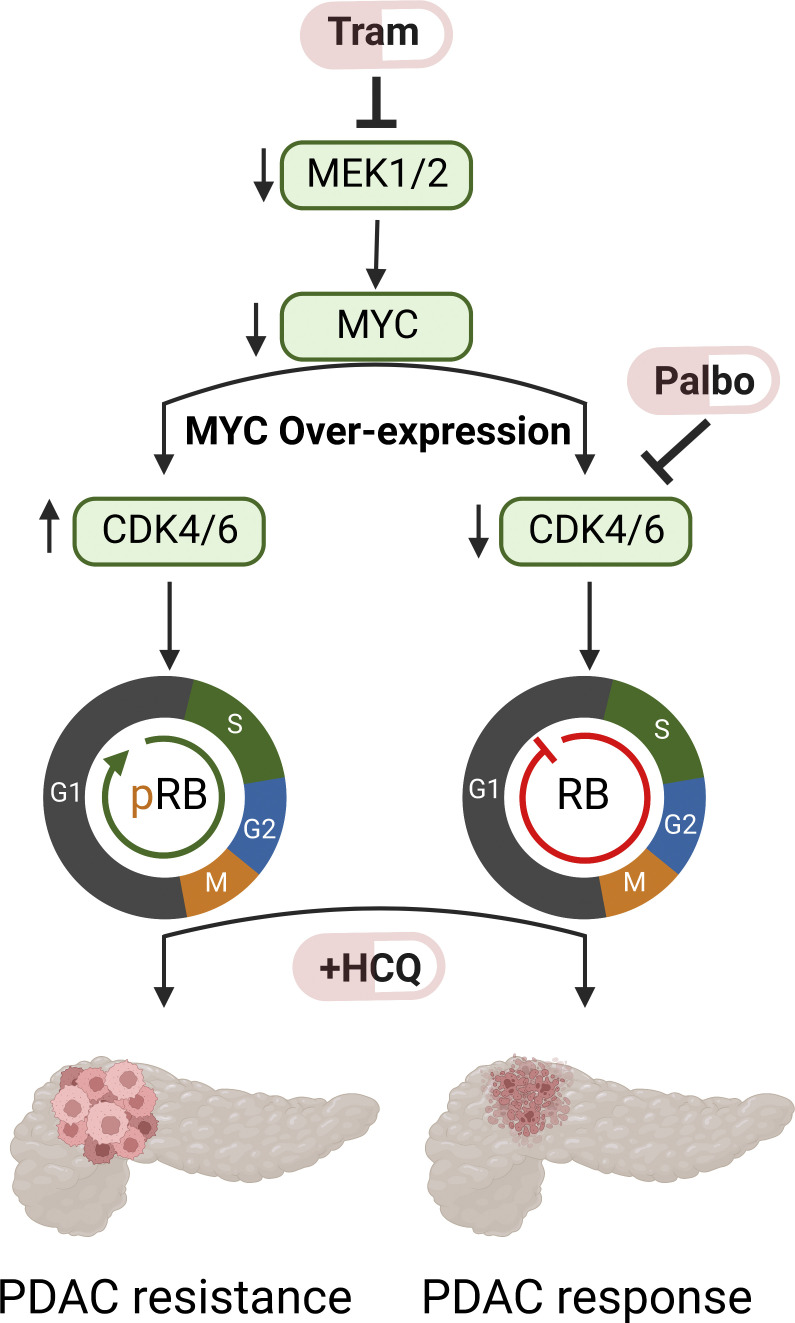

## Introduction

Pancreatic ductal adenocarcinoma (PDAC) is an aggressive and recalcitrant malignancy for which innovative therapeutic options that improve both the duration and quality of PDAC patients’ lives are urgently required. Indeed, PDAC is predicted to become the second most common cause of cancer death in 2030 (Rahib et al., 2014). PDAC is frequently detected at an advanced stage when it is unresectable and often recurs despite curative intent by surgical resection, making systemic therapies the mainstay of treatment.

PDAC is driven by mutationally activated KRAS, which is detected in >90% of PDACs (Kanda et al., 2012). Oncogenic KRAS contributes to PDAC pathology by promoting malignant transformation, tumor maintenance, metabolic rewiring, metastasis, desmoplasia, and immune cell dysregulation (Waters and Der, 2018). KRAS oncoproteins signal through multiple downstream effectors including the MAPK and PI3K pathways. However, to date, pathway-targeted therapies that inhibit these signaling pathways have not improved patient survival (Roth et al., 2020). For example, inhibition of MAPK (RAF>MEK1/2>ERK1/2) signaling with trametinib, combined with gemcitabine, failed to improve overall or progression-free survival, overall response rate, or duration of response in untreated metastatic PDAC patients (Infante et al., 2014). Although mutant KRAS inhibitors are in development, these are still under pre-clinical and early clinical investigation (Janes et al., 2018; Hong et al., 2020; Wang et al., 2022). Thus, it remains to be seen whether targeting KRAS oncoproteins or downstream pathways will benefit PDAC patients.

KRAS-driven PDAC cells display elevated macroautophagy (autophagy), a recycling process that targets intracellular components for lysosome-mediated degradation providing metabolic substrates required for the high metabolic demand of rapidly proliferating cells (Yang et al., 2011). However, preventing lysosomal degradation with hydroxychloroquine (HCQ) failed to provide clinical benefit to PDAC patients (Wolpin et al., 2014). We and others demonstrated that inhibition of KRAS>RAF>MEK1/2>ERK1/2 signaling resulted in the induction of cytoprotective autophagy through the LKB1>AMPK>ULK1 signaling axis (Kinsey et al., 2019; Bryant et al., 2019). Moreover, combining a MEK1/2 inhibitor (trametinib [T]) plus an autophagy inhibitor (chloroquine [CQ] or HCQ) displayed cytotoxic synergy in vitro and regression of xenografted PDAC tumors in mice. Moreover, T/HCQ treatment of a PDAC patient with recurrent, metastatic disease elicited a partial, but striking response (Kinsey et al., 2019). Thus, several clinical trials were initiated to determine the safety and preliminary efficacy of MAPK pathway inhibitors combined with HCQ (clinicaltrials.gov: NCT04214418, NCT04132505, NCT04386057, NCT04145297). Further to our patient case, others reported disease stabilization after T/HCQ treatment in two patients with chemoresistant PDAC (Xavier et al., 2020). Despite the potential therapeutic benefit of T/HCQ treatment, we report here the natural histories of three heavily pre-treated PDAC patients who exhibited intrinsic or acquired resistance to T/HCQ, prompting us to explore how T/HCQ therapy resistance is mediated, and investigate a therapeutic strategy to combat resistant disease.

The *c-MYC* proto-oncogene is commonly amplified in human cancer, with ∼50% of all cancers displaying elevated expression of c-MYC (Vita and Henriksson, 2006). c-MYC expression is generally low in the normal adult pancreas but genomic copy number gains encompassing the *c-MYC* locus on chromosome 8q24 and oncogenic KRAS-induced c-MYC overexpression are detected in ∼40% of human PDACs (Mahlamaki et al., 2002; Schleger et al., 2002). In addition, c-*MYC* amplification correlates with a poorer prognosis for PDAC patients (He et al., 2014). Furthermore, dysregulation of c-MYC mediates drug resistance in PDAC (Parasido et al., 2019). Here, we report high c-MYC expression in PDACs that have primary or acquired chemoresistance to T/HCQ therapy. Furthermore, we demonstrate that the ectopic expression of c-MYC in human PDAC cells promotes cell cycle re-entry rendering them resistant to T/CQ treatment. Additionally, we show induction of protective autophagy in PDAC cell lines after treatment with CDK4/6 inhibitors (e.g., palbociclib [P]), which synergizes with CQ to induce stasis of xenografted tumors, even in the face of c-MYC overexpression. Strikingly, P/HCQ treatment resulted in a substantial decrease in the blood-borne cancer antigen 19-9 (CA19-9) biomarker, with correlative evidence of tumor lysis in a PDAC patient who developed resistant metastases following an initial response to T/HCQ therapy (Kinsey et al., 2019). Thus, we propose that elevated c-MYC expression is both a biomarker and a mediator of T/HCQ resistance, and further propose to evaluate P/HCQ as a potential treatment option to treat T/HCQ-resistant PDAC.

## Results

### Elevated c-MYC expression is detected in PDACs that display innate or acquired resistance to trametinib plus HCQ

**Figure 1.**
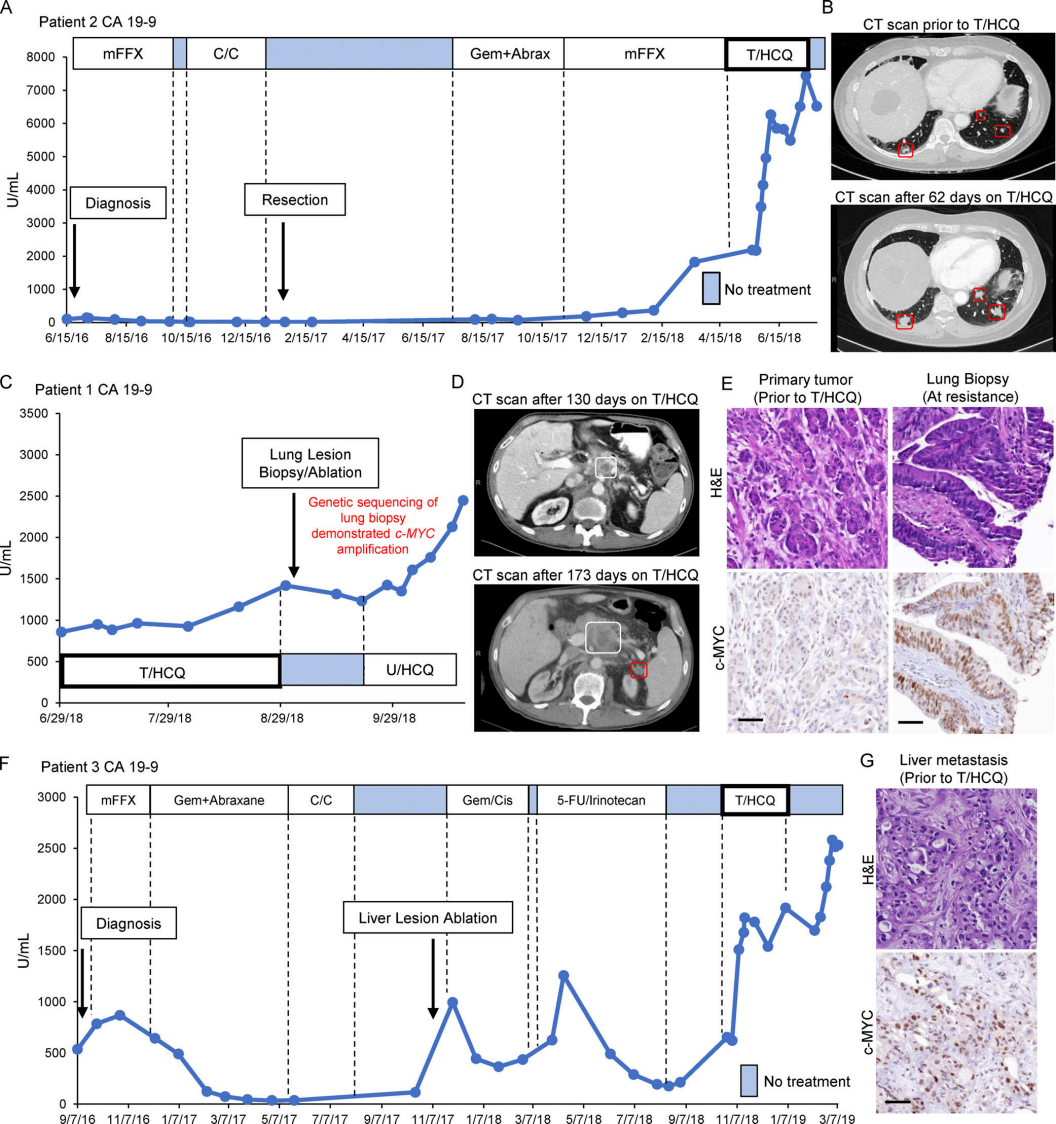
**c-MYC expression is elevated in PDACs that display innate or acquired resistance to T/HCQ. (A)** Patient 2’s CA19-9 levels are displayed and annotated with the dates and treatments administered throughout the clinical course. CA19-9 levels rose despite initiation of 2 mg trametinib (q.d.) combined with an escalating dose (increased every 3 d) of HCQ, starting at 400 mg (q.d.) and plateauing at 1,200 mg (600 mg b.i.d.). Initial diagnosis and primary tumor resection are indicated with arrows. Blue boxes indicate treatment cessation. mFFX, mFOLFIRINOX; C/C, chemorads with capecitabine; Gem+Abrax, gemcitabine and abraxane. **(B)** CT imaging for Patient 2 1 d prior to starting T/HCQ (top), and after 62 d of T/HCQ treatment (bottom). The metastatic lung lesions (red boxes) increase in size, which is consistent with progressive disease on T/HCQ treatment. **(C)** CA19-9 levels and treatment course (continued from Kinsey et al., 2019) for Patient 1 are displayed. *c-MYC* amplification was observed in genetic sequencing of a lung lesion biopsy obtained after resistance occurred. U/HCQ, ulixertinib and HCQ. **(D)** CT imaging for Patient 1 after 130 d on T/HCQ treatment (top), and after 173 d on T/HCQ treatment (bottom). The primary pancreatic lesion (highlighted in white) increased in size, and a new peritoneal lesion was detected (highlighted in red), consistent with progressive disease on T/HCQ treatment. **(E)** Representative IHC images of tissue samples obtained from Patient 1’s T/HCQ-resistant lung metastasis revealed increased c-MYC expression relative to Patient 1’s primary tumor. Top panels show H&E staining; bottom panels are representative images of c-MYC IHC. Scale bars are 50 μm. **(F)** Patient 3’s CA19-9 levels are displayed throughout clinical course. CA19-9 increased despite initiation of 2 mg trametinib (q.d.) combined with HCQ, starting at 400 mg (q.d.), and reaching a maximum of 1,200 mg (600 mg b.i.d.). Initial diagnosis and liver lesion ablation are indicated with arrows, and blue boxes indicate treatment cessation. Gem/Cis, gemcitabine and cisplatin; 5-FU/Irinotecan, fluorouracil and irinotecan. **(G)** Representative IHC of a liver metastasis obtained from Patient 3 prior to T/HCQ treatment revealed elevated c-MYC expression. Top panel shows H&E staining; bottom panel is a representative image of c-MYC IHC. Scale bar is 50 μm.

We previously reported that the partial response of a PDAC patient with metastatic disease (Patient 1) upon treatment with trametinib (2 mg/d) combined with HCQ (1,200 mg/d, T_2_/HCQ_1200_; Kinsey et al., 2019). Based on preclinical data describing the anti-tumor effect of T/HCQ and Patient 1’s response, we treated a second patient (Patient 2) whose disease had also progressed through all standard-of-care PDAC therapies (Fig. 1 A). Patient 2 was treated off-label/off-trial with trametinib (2 mg/d) plus HCQ (400 mg/d; T_2_/HCQ_400_) with dose escalation to 800 mg/d, and then 1,200 mg/d (T_2_/HCQ_1200_) to allow for potential toxicities to become apparent and mitigated. Unfortunately, Patient 2’s CA19-9 levels continued to rise (Fig. 1 A) and computed tomography (CT) imaging after 2 mo of T_2_/HCQ_1200_ therapy showed progressive disease consistent with intrinsic resistance to T/HCQ treatment (Fig. 1 B). Comparison of the genetic abnormalities revealed by sequencing somatic tumor DNA (Tempus Oncology) from Patients 1 and 2 indicated that, while both tumors had mutations in *KRAS* and *TP53*, Patient 2's tumor had a focal DNA copy number gain encompassing *c-MYC* on chromosome 8. Moreover, Patient 1, who initially responded to T_2_/HCQ_1200_ treatment (Kinsey et al., 2019), eventually acquired resistance as indicated by escalating CA19-9 levels and progressive disease on CT imaging (Fig. 1, C and D). A T/HCQ-resistant lung metastasis was subsequently biopsied for (1) immunohistochemical (IHC) analysis, (2) generation of a patient-derived xenograft (PDX), and (3) analysis of somatic tumor DNA. Interestingly, this metastasis also displayed a focal copy number gain encompassing *c-MYC* (Foundation One). Furthermore, IHC analysis revealed elevated c-MYC expression in the T/HCQ-resistant metastasis relative to the primary lesion obtained during Patient 1’s initial surgical resection (Fig. 1 E). Finally, analysis of a liver metastasis biopsy taken prior to T/HCQ treatment detected elevated c-MYC expression in a third patient (Patient 3), who also harbored T/HCQ-resistant disease (Fig. 1, F and G). Taken together, these data suggested a correlation between elevated c-MYC and resistance to T/HCQ.

### c-MYC confers resistance to combined trametinib plus CQ treatment in PDAC

**Figure S1.**
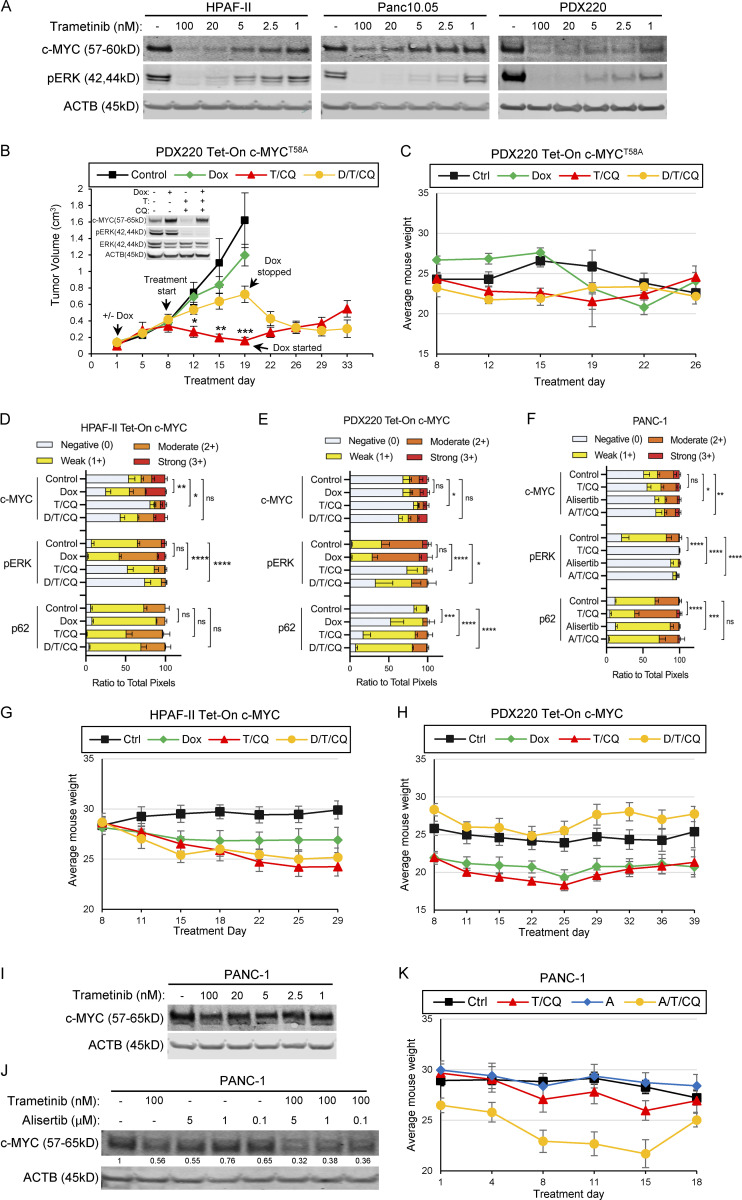
**Trametinib reduces c-MYC expression in human PDAC cells.** **(A)** HPAF-II, Panc 10.05, and PDX220 lysates were blotted for c-MYC, pERK1/2, and ACTB after treatment with the indicated concentrations of trametinib for 48 h (*n* = 1 for HPAF-II and Panc 10.05, *n* = 2 for PDX220). **(B and C)** c-MYC^T58A^ expression mediates T/HCQ resistance in PDAC. The tumor volumes of PDX220 expressing Tet-On c-MYC^T58A^ are shown. Inset depicts representative immunoblot analysis of c-MYC, pERK1/2, ERK1/2, and ACTB in cells before injection into mice. Cells were treated with DMSO (first column), 2 μg/ml Dox, 100 nM trametinib (T), and 20 μM CQ, or the combinations thereof, for 48 h. Tumor-bearing mice were treated with: (1) corn oil (control, *n* = 6); (2) 625 mg/kg Dox chow (*n* = 6); (3) 1 mg/kg trametinib and 50 mg/kg CQ (T/CQ, *n* = 6); or (4) the triple combination of Dox, trametinib, and CQ (D/T/CQ, *n* = 6), where *n* equals the number of mice. Tumor volumes were assessed over 33 d and arrows denote start of treatments, including when Dox was started or stopped after treatment initiation. Lines are the mean tumor volumes ± SEM. *, P < 0.01; **, P < 0.005; ***, P < 0.0005 by Student’s two-sided *t* test. Average mouse weights for each treatment group with xenografted PDX220 Tet-On c-MYC^T58A^ are shown in C. Error bars represent SEM. **(D–F)** Quantitation of IHC performed on xenografts shown in Fig. 2, taken from three regions of stained tumor sections. ns, not significant; *, P < 0.05; **, P < 0.005; ***, P < 0.0005; ****, P < 0.0001 by two-way ANOVA. **(G and H)** Mouse weights for each xenografted cell type (G, HPAF-II Tet-On c-MYC; H, PDX220 Tet-On c-MYC) were averaged. Error bars represent SEM. **(I and J)** Alisertib combined with trametinib reduces c-MYC expression in PANC-1 cells. PANC-1 lysates were blotted for c-MYC and ACTB after treatment with trametinib and/or alisertib for 48 h at the indicated concentrations. Representative blots from two (for I) and five (for J) independent experiments are shown. **(K)** Mouse weights of xenografted PANC-1 were averaged. Error bars represent SEM. Source data are available for this figure: SourceData FS1.

**Figure 2.**
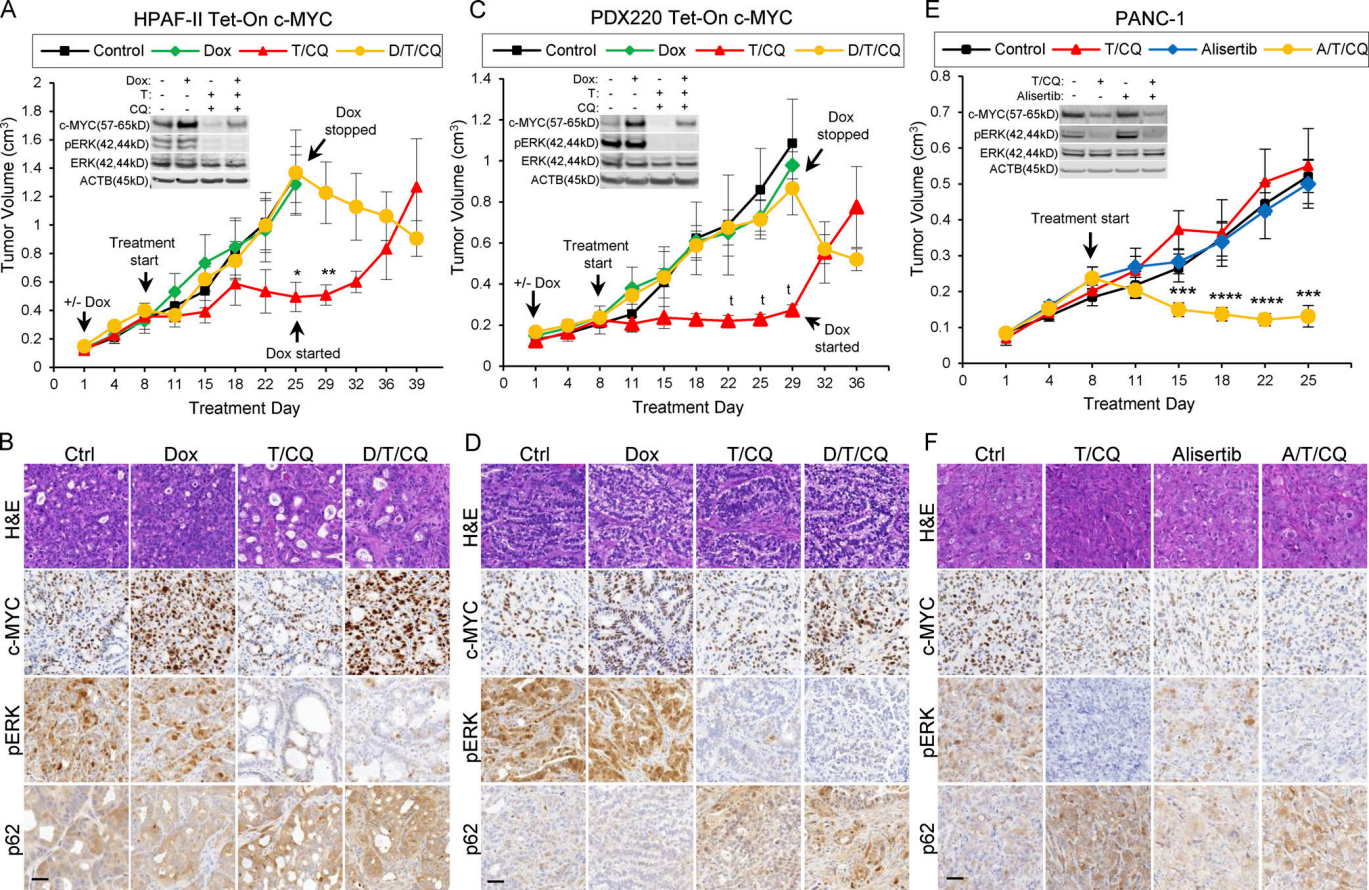
**c-MYC determines sensitivity to T/CQ in xenografted pancreatic tumors. (A and C)** Tumor volumes of a xenografted human PDAC cell line (HPAF-II; A) and a PDX (PDX220; C) expressing Dox-regulated c-MYC (Tet-On c-MYC) are shown. Insets depict representative immunoblot analysis of cells before injection into NOD/SCID mice. Cells were treated with DMSO (first column), 2 μg/ml Dox, 100 nM trametinib (T), and 20 μM CQ, or the combinations thereof, for 48 h. Tumor-bearing mice were treated with: (1) corn oil (control, *n* = 3, black line); (2) 625 mg/kg Dox chow (*n* = 4, green line); (3) 1 mg/kg trametinib and 50 mg/kg CQ (T/CQ, *n* = 6, red line); or (4) doxycycline, trametinib, and CQ (D/T/CQ, *n* = 5, yellow line), where *n* equals the number of mice. Arrows denote start of treatments, including when Dox was started or stopped. Lines are the mean tumor volumes ± SEM. *, P < 0.05; **, P < 0.01; ^t^, P < 0.0005; Student’s two-sided *t* test. **(B and D)** Representative images of IHC analysis on xenografted HPAF-II or PDX220 Tet-On c-MYC tumors from mice treated as in A and C. Sections were stained with H&E or with indicated antibodies. Scale bars are 50 μm. **(E)** Tumor volumes of xenografted PANC-1. Tumor-bearing mice were treated with: (1) corn oil (control, *n* = 6); (2) 20 mg/kg alisertib (Alisertib, *n* = 6); (3) trametinib and CQ (T/CQ, *n* = 6, T: 1 mg/kg and CQ: 50 mg/kg); or (4) the triple combination (A/T/CQ, *n* = 5, yellow line), where *n* equals the number of mice. Lines represent the mean ± SEM. ***, P < 0.005; ****, P < 0.001; Student’s two-sided *t* test. **(F)** Representative images of IHC on xenografted PANC-1 tumors from mice treated as in E. Sections were stained with H&E or with indicated antibodies. Scale bar represents 50 μm. Source data are available for this figure: SourceData F2.

**Figure S2.**
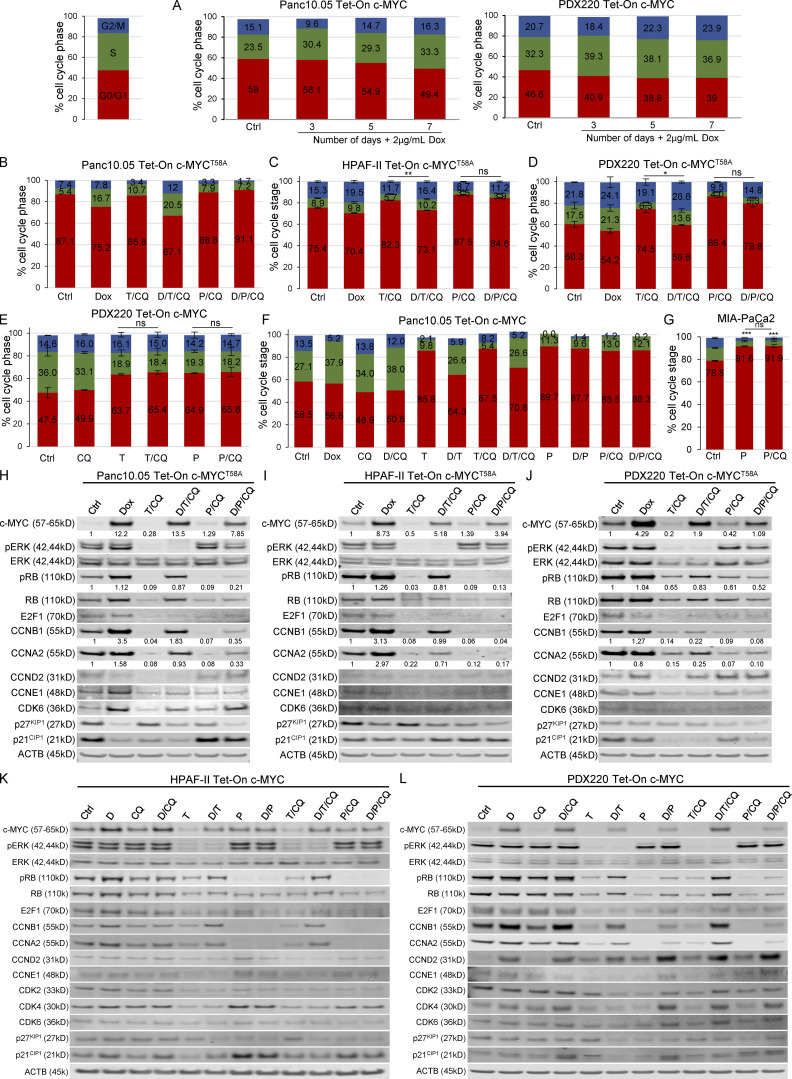
**c-MYC expression drives cell cycle progression. (A)** Tet-On c-MYC–expressing PDX220 or Panc 10.05 cells treated with vehicle or 2 μg/ml Dox for 3, 5, or 7 d were assessed for cell cycle. Bar graphs show representative data from two independent experiments for PDX220 and one experiment for Panc 10.05. **(B–D)** c-MYC^T58A^ reactivates the cell cycle in the face of T/CQ co-treatment, but not P/CQ co-treatment. Percentage of cells in each cell cycle phase in (B) Panc 10.05 (*n* = 1 from one independent experiment), (C) HPAF-II (*n* = 3 from one experiment), or (D) PDX220 cells (*n* = 1 from three independent experiments) expressing Tet-On c-MYC^T58A^ are depicted. Cells were treated for 7 d with DMSO (Ctrl), 2 μg/ml Dox, 10–100 nM trametinib and 10–20 μM CQ (T/CQ), D/T/CQ, 2.5 μM palbociclib and 10–20 μM CQ (P/CQ) or D/P/CQ and cell cycle phase was quantitated. Bars represent mean percent cell cycle phase ± SEM. *, P < 0.05; **, P < 0.05; ns, not significant by Student’s two-sided *t* test. **(E)** CQ co-treatment does not significantly affect cell cycle status in trametinib- or palbociclib-treated PDAC cells. PDX220 Tet-On c-MYC cells were treated with vehicle control (Ctrl), CQ (20 μM), trametinib (T, 100 nM), palbociclib (P, 2.5 μM), T/CQ, or P/CQ for 7 d and processed for cell cycle analysis (*n* = 1 from two independent experiments, mean ± SEM). ns, not significant by Student’s two-tailed *t* test. **(F)** Panc 10.05 cells expressing Tet-On c-MYC were treated for 7 d with DMSO (Ctrl), CQ (20 μM), trametinib (T, 100 nM), or palbociclib (P, 2.5 μM) or the combinations (T/CQ and P/CQ), and with or without Dox (D, 2 μg/ml; *n* = 1). **(G)** Cell cycle profiles were assessed in MIA-PaCa2 cells after treatment with palbociclib with and without CQ co-treatment. A Student’s two-tailed *t* test was utilized in order to determine statistical significance of G_0_/G_1_ phase between the DMSO control and the experimental groups as well as between the experimental groups in one experiment (*n* = 3). ns, not significant; ***, P < 0.001. **(H–J)** c-MYC^T58A^ expression elevates pRB, cyclinB1, and cyclinA2 expression in the face of trametinib and CQ, but not P/CQ co-treatment. Representative immunoblot analysis of Tet-On c-MYC^T58A^ expressing Panc 10.05 (H), HPAF-II (I), and PDX220 (J) cells that were treated as in B–D (from three independent experiments). Lysates were blotted for c-MYC, pERK1/2, ERK1/2, pRB, RB, E2F1, CCNB1, CCNA2, CCND2, CCNE1, CDK6, p27^KIP1^, p21^CIP1^, and ACTB. **(K and L)** c-MYC^WT^ elevates pRB, cyclinB1, and cyclinA2 expression in the face of T/CQ, but not P/CQ. Representative immunoblot analysis of Tet-On c-MYC HPAF-II and PDX220 cells that were treated as above (from two independent experiments for HPAF-II and *n* = 1 experiment for PDX220). Lysates were blotted for c-MYC, pERK1/2, ERK1/2, pRB, RB, E2F1, CCNB1, CCNA2, CCND2, CCNE1, CDK2, CDK4, CDK6, p27^KIP1^, p21^CIP1^, and ACTB. Source data are available for this figure: SourceData FS2.

**Figure 3.**
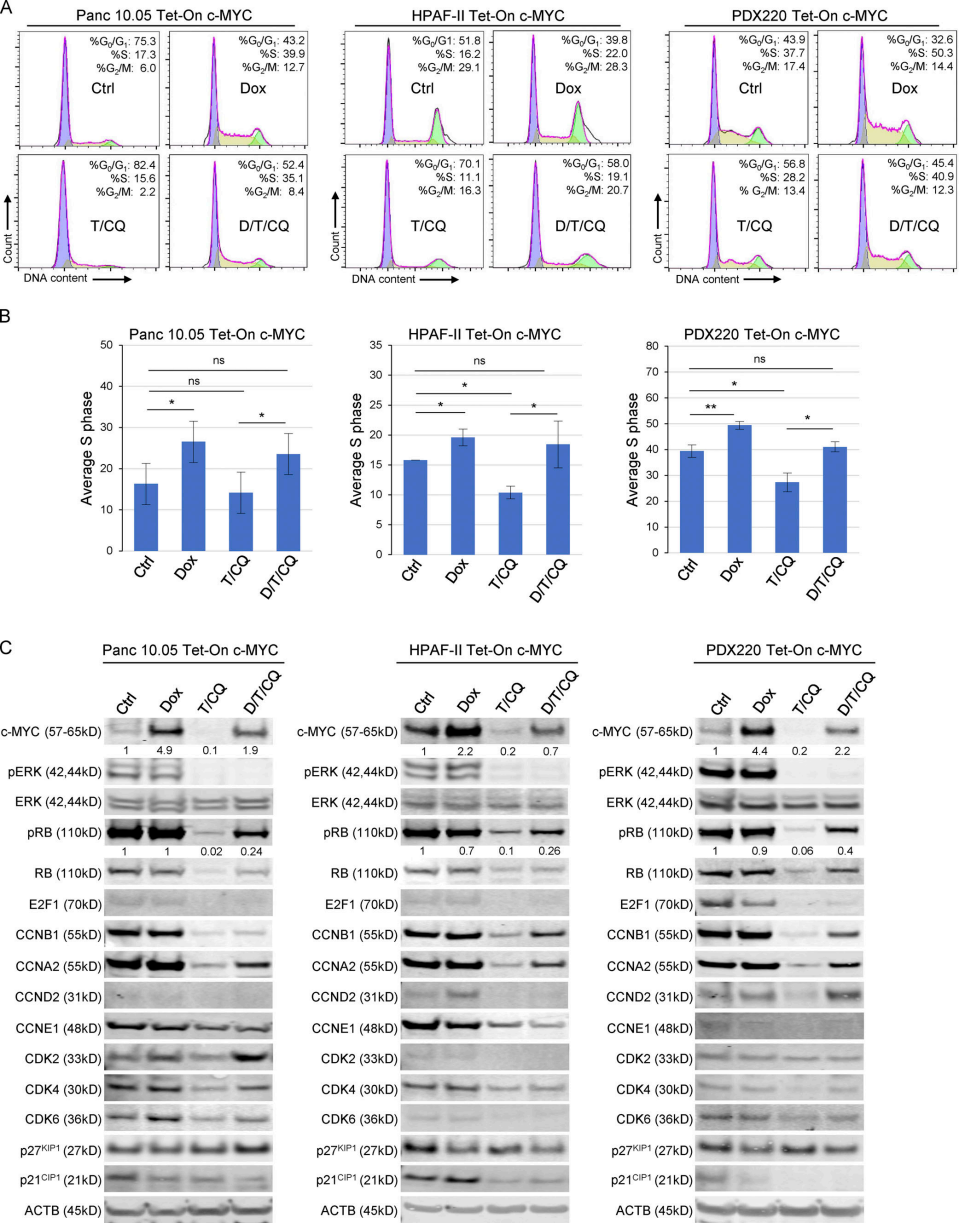
**Elevated c-MYC expression prevents T/CQ-mediated cell cycle arrest. (A)** Representative cell cycle histograms with percentages of cells in G_0_/G_1_, S, and G_2_/M phases are shown for Panc 10.05, HPAF-II, and PDX220 cells expressing Tet-On c-MYC. Cells were treated for 7 d with DMSO (Ctrl), 2 μg/ml Dox, trametinib (T, at 5–100 nM) and CQ (at 5–20 μM; T/CQ), or D/T/CQ. **(B)** Average percentage of cells in S phase for Tet-On c-MYC–expressing Panc 10.05, HPAF-II, and PDX220 cells that were treated as above (*n* = 3 from two independent experiments for each cell type, mean ± SEM). *, P < 0.05; **, P < 0.01; Student’s two-sided *t* test. **(C)** Immunoblot analysis of lysates generated from Tet-On c-MYC–expressing cells that were treated as above (*n* = 1 for each cell type). Lysates were blotted for c-MYC, pERK1/2 (phospho-T202/Y204), ERK1/2, pRB (phospho-S807/S811), RB, E2F1, CCNB1, CCNA2, CCND2, CCNE1, CDK2, CDK4, CDK6, p27^KIP1^, p21^CIP1^, and ACTB. Densitometry quantitation of c-MYC and pRB protein levels normalized to actin are shown. Source data are available for this figure: SourceData F3.

Mutationally activated KRAS elevates c-MYC expression through its canonical effectors, RAF and PI3K (Land et al., 1983; Sears et al., 2000). Conversely, the inhibition of MAPK signaling inhibits c-MYC expression in some PDAC models (Gysin et al., 2012; Hayes et al., 2016). Human PDAC cell lines HPAF-II and Panc 10.05 express c-MYC that is sensitive to MEK1/2 inhibition (Vaseva et al., 2018), and we observed the same effect in a PDX-derived cell line, PDX220 (Fig. S1 A; Kinsey et al., 2019). To test whether the elevated c-MYC expression renders PDAC cells resistant to T/HCQ, we engineered PDAC cell lines to conditionally express c-MYC^WT^ or c-MYC^T58A^, the latter a stabilized form of c-MYC. Doxycycline (Dox) treatment resulted in an approximately two- to five-fold increase in c-MYC expression relative to endogenous protein (insets in Fig. 2, A and C; and Fig. S1 B). As expected, trametinib treatment reduced c-MYC expression, and co-treatment with CQ had no further effect (Fig. S2, K and L). Importantly, the addition of Dox to T/CQ-treated cells restored c-MYC expression back to ∼0.7- to 2.2-fold of control levels (quantitated in Fig. 3 C). To assess whether ectopic c-MYC expression mediated resistance to T/CQ in vivo*,* HPAF-II or PDX220 cells expressing Tet-On c-MYC were xenografted into NOD/SCID mice. While tumor-bearing mice fed control chow (−Dox) demonstrated tumor stasis or regression with T/CQ treatment, ectopic expression of either c-MYC^WT^ (in HPAF-II or PDX220; Fig. 2, A and C) or c-MYC^T58A^ (in PDX220; Fig. S1 B) in mice fed Dox containing (+Dox) chow rendered tumors resistant to T/CQ treatment. Furthermore, when mice treated with T/CQ were fed Dox chow to induce c-MYC expression in tumors that had initially responded to T/CQ treatment, tumor regrowth was observed, suggesting that c-MYC expression can mediate acquired T/HCQ resistance. Conversely, removal of Dox chow from mice with initially resistant disease resulted in tumor regression, suggesting that reducing c-MYC expression sensitizes tumors to T/CQ treatment. Tumors in T/CQ treatment groups displayed reduced c-MYC and pERK, while co-treatment with Dox restored c-MYC expression (Fig. 2, B and D; and Fig. S1, D and E).

We hypothesized that the reduced c-MYC expression in T/CQ-resistant cells might sensitize such cells to T/CQ treatment. Inhibitors of Aurora kinase A (AURKA), such as alisertib, destabilize c-MYC by nullifying the protection from FBW7-mediated proteasomal degradation provided by AURKA when bound to c-MYC (Dauch et al., 2016). Consistent with published results, we observed that c-MYC expression was resistant to trametinib in PANC-1 cells (Fig. S1 I; Hayes et al., 2016). As expected, alisertib treatment of PANC-1 cells resulted in reduced c-MYC expression, which was further enhanced by the addition of trametinib (Fig. 2 E, inset; and Fig. S1 J). Next, mice-bearing PANC-1 tumors were treated with (1) vehicle, (2) combination of T/CQ, (3) alisertib alone, or (4) alisertib plus T/CQ. PANC-1 tumors were resistant to the T/CQ combination or alisertib monotherapy. However, alisertib plus T/CQ elicited tumor regression, suggesting that reducing the c-MYC expression by AURKA blockade conferred sensitivity to T/CQ therapy. Tumors in the T/CQ and alisertib treatment groups displayed reduced c-MYC expression, but an additional decrease in c-MYC was detected in alisertib plus T/CQ treatment group (Fig. 2 F and Fig. S1 F). As anticipated, trametinib treatment resulted in reduced pERK1/2, while CQ treatment resulted in an increase in p62^SQSTM1^. Together, these studies demonstrate that the elevated c-MYC expression confers resistance to T/CQ treatment and suggest that reducing c-MYC expression may increase sensitivity in the T/CQ-resistant disease.

### c-MYC promotes cell cycle progression in the face of combined treatment with trametinib plus CQ

To explore how c-MYC mediates resistance to T/CQ, we examined the cell division cycle, one of the hallmarks of cancer modulated by activated KRAS and c-MYC (Gysin et al., 2005; Gysin et al., 2012; Hanahan and Weinberg, 2000). To test whether elevated c-MYC expression overrides T/CQ-induced cell cycle arrest, we analyzed cell cycle phases in PDAC cell lines in the absence (–Dox) or presence (+Dox) of ectopic c-MYC. Relative to controls, c-MYC expression increased the percentage of cells in S or G_2_/M phase, with a ∼5–10% increase in S phase evident after 7 d of ectopic c-MYC expression (Fig. 3, A and B; and Fig. S2, A–D). As expected, T/CQ treatment reduced the percentage of cells in the S or G_2_/M phases, but the expression of either c-MYC^WT^ (Fig. 3, A and B; and Fig. S2 F) or c-MYC^T58A^ (Fig. S2, B–D) overcame this cell cycle arrest and restored the percentage of S phase cells to control levels. Consequently, these results demonstrate that over-expression of c-MYC overcomes the cell cycle arrest that occurs after T/CQ treatment in vitro.

To characterize the effects of c-MYC expression on the cell cycle machinery, we conducted immunoblot analysis of cell cycle regulators that are transcriptionally controlled by c-MYC (Bretones et al., 2015). The c-MYC^WT^ expression had little effect on baseline expression of cyclins or CDKs (cyclin-dependent kinases; Fig. 3 C), but c-MYC^T58A^ increased cyclins A2 and B1 expression in HPAF-II and Panc 10.05 cells (Fig. S2, H and I). However, c-MYC^T58A^ was expressed at ∼8- to 12-fold over endogenous c-MYC while ectopic c-MYC^WT^ expression was two- to five-fold higher, which may account for the differential effects observed on cyclin expression by c-MYC^T58A^. In addition, c-MYC^T58A^ may be altered in its ability to regulate c-MYC target genes (Hemann et al., 2005). c-MYC^WT^ expression led to decreased p27^KIP1^ expression, consistent with previous reports (Fig. 3 C, HPAF-II and PDX220; Bretones et al., 2015). Both T and T/CQ treatments reduced retinoblastoma protein (RB) phosphorylation and the total expression of RB, E2F1, A-, B-, D-, and E-type cyclins, CDK2, 4, and 6, and p21^CIP1^ (Fig. 3 C and Fig. S2, H–L). Interestingly, the expression of either c-MYC^WT^ or c-MYC^T58A^ in trametinib or T/CQ-treated PDAC cells elicited RB phosphorylation (pS807/pS811; Fig. 3 C and Fig. S2, H–L). Unexpectedly, c-MYC did not induce the expression of CDK2 or cyclin E1 (Perez-Roger et al., 1997). However, the c-MYC expression in trametinib or T/CQ-treated cells increased cyclins A2 and B1 expression. Cyclins A2 and B1 are essential for cell cycle completion in the minimal model of cell cycle (Hochegger et al., 2008; Murphy et al., 1997; Brandeis et al., 1998), and cyclin A is the primary cyclin to facilitate c-MYC–driven cell cycle progression in lung cell lines (Qi et al., 2007), which is consistent with our observations. These findings suggest that c-MYC–mediated effects on cyclins A2 and B1, RB inactivation, and p27^KIP1^ reduction are sufficient to promote cell cycle progression in the presence of T/CQ. Together, these data demonstrate that induced c-MYC expression overrides T/CQ-induced cell cycle arrest.

### Pharmacological inhibition of CDK4/6 induces autophagy in PDAC cell lines

**Figure 4.**
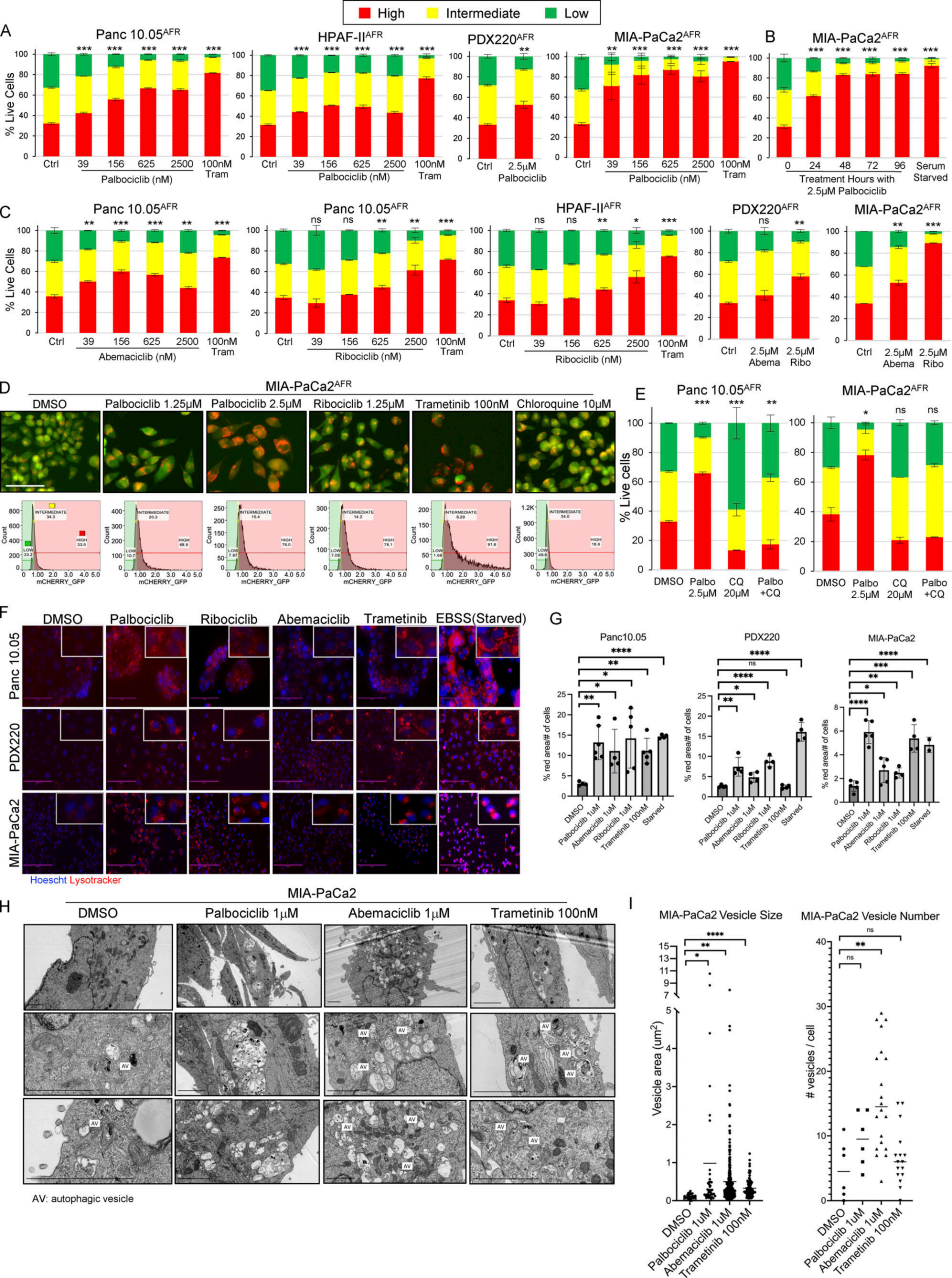
**CDK4/6 inhibition induces autophagy in human PDAC lines. (A–C)** Panc 10.05, HPAF-II, PDX220, and MIA-PaCa2 expressing the AFR were treated with palbociclib, ribociclib, or abemaciclib at the indicated concentrations for 48 h unless otherwise noted. An increased ratio of mCherry:eGFP within the high autophagy gate indicates elevated autophagic flux. Center values are the mean and error bars are SD from one experiment (*n* = 3) except for MIA-PaCa2 cells treated with palbociclib, which were from two experiments (*n* = 3). A Student’s two-tailed *t* test was utilized to determine statistical significance between the high autophagy gates for each control and experimental group. ns, not significant; *, P < 0.05; **, P < 0.01; ***, P < 0.001. **(D)** Fluorescent images (mCherry and eGFP merged) of MIA-PaCa2^AFR^ cells after treatment (top) and representative flow cytometry histograms (bottom) show gates for mCherry:eGFP ratiometric quantitation of low (green), intermediate (yellow), and high (red) autophagic flux. Scale bar is 100 μm. **(E)** MIA-PaCa2 cells expressing the AFR were treated with palbociclib, CQ, or P/CQ, and autophagic flux was assessed. (Panc 10.05, *n* = 3; MIA-PaCa2, *n* = 2; mean values ± SD from one experiment.) *, P < 0.05; **, P < 0.01; ***, P < 0.001. A Student’s two-tailed *t* test was utilized to determine statistical significance of the high autophagy gate between the control groups and experiment groups. **(F and G)** PDAC cell lines were treated with CDK4/6 inhibitors, trametinib, or EBSS. After 48 h, cells were incubated with Hoescht and Lysotracker. Scale bar is 100 μm. A Student’s two-tailed *t* test was used to determine statistical significance between the DMSO control and each experimental group from two experiments (*n* = 2); *, P < 0.05; **, P < 0.01; ***, P < 0.001; ****, P < 0.0001. **(H and I)** MIA-PaCa2 were treated with palbociclib, abemaciclib, or trametinib for 48 h before processing for electron microscopy. The number of single and/or double membraned vesicles per cell and the vesicle size were quantified. A Student’s two-tailed *t* test was used to determine statistical significance between the DMSO control and each experimental group from one experiment (*n* = 1); *, P < 0.05; **, P < 0.01; ****, P < 0.0001. Scale bars in each image are 2 μm.

KRAS-regulated downstream signaling pathways promote G_0_>G_1_>S-phase cell cycle transitions through regulation of D-type cyclins and their partners, CDK4 and CDK6 (CDK4/6; Lavoie et al., 1996; Cheng et al., 1998). Hence, concurrently, we were evaluating the ability of CDK4/6 inhibitors to modulate autophagy in PDAC cell lines. Indeed, prior literature suggested a relationship between cell cycle arrest and autophagy induction (Liang et al., 2007; Capparelli et al., 2012; Lipinski et al., 2010). To determine whether CDK4/6 inhibition modulates autophagy, we treated four PDAC cell lines expressing the mCherry-eGFP-LC3B autophagic flux reporter (AFR) with palbociclib (Fig. 4, A and B) and observed an induction of autophagy compared to control (Gump and Thorburn, 2014; Kinsey et al., 2019). To test whether autophagy is induced by CDK4/6 inhibition rather than an off-target effect of palbociclib, PDAC cells were treated with the additional CDK4/6 inhibitors (abemaciclib or ribociclib), which again induced autophagy (Fig. 4 C). Fluorescent images of live MIA-PaCa2^AFR^ cells treated with palbociclib, ribociclib, or trametinib demonstrated the expected decrease in eGFP positive punctae compared to DMSO control (Fig. 4 D). Additionally, we observed that the CDK4/6 inhibition with palbociclib, abemaciclib, or ribociclib resulted in increased acidic organelles (Fig. 4, F and G), to a similar extent as cells treated with trametinib or Earle’s Balanced Salt Solution (EBSS; starvation control). To further characterize these organelles, we conducted electron microscopy and observed that treatment with palbociclib or abemaciclib resulted in a significant increase in the size of single- and/or double-membraned autophagic vesicles (Fig. 4, H and I). Surprisingly, although treatment with abemaciclib resulted in a significant increase in vesicle number, palbociclib and trametinib treatment did not. Finally, CQ-mediated inhibition of lysosome-autophagosome fusion inhibited palbociclib-induced autophagy in Panc 10.05^AFR^ and MIA-PaCa2^AFR^ cells (Fig. 4 E).

### Palbociclib plus CQ delays PDAC growth in vitro

**Figure 5.**
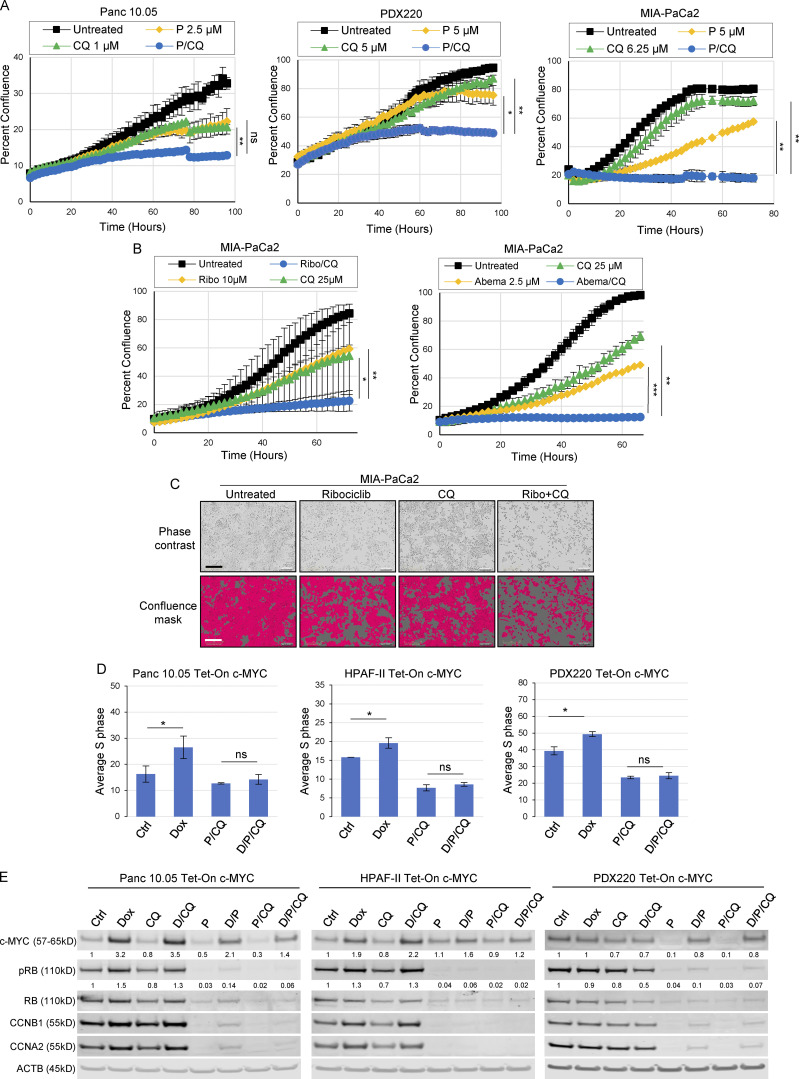
**Elevated c-MYC expression does not prevent P/CQ-mediated cell cycle arrest. (A)** Panc 10.05, PDX220, and MIA-PaCa2 cells were imaged to assess confluence over time using the IncuCyte Live-Cell Analysis System after treatment with palbociclib (P), CQ, or the combination of both agents (P/CQ). Data represent mean values, and error bars reflect SD of one experiment (*n* = 3). One-way ANOVA was conducted to determine statistical difference between the single agent controls (CDK4/6 inhibitor or CQ) and the combination group. *, P < 0.05; **, P < 0.01; ***, P < 0.001. **(B)** MIA-PaCa2 cells were treated with ribociclib (Ribo) or abemaciclib (Abema) alone or in combination with CQ and confluence was measured over time. A one-way ANOVA was utilized to determine statistical significance between the single agent controls and the combination group. The Ribo/CQ experiment was conducted twice (*n* = 2) and the Abema/CQ experiment was conducted once (*n* = 3). *, P < 0.05; **, P < 0.01; ***, P < 0.001. **(C)** Representative images of MIA-PaCa2 cells shown by phase contrast (top) and the corresponding cell mask utilized to estimate well confluence (bottom) for each treatment at 48 h. Bars are 300 μm. **(D)** Average percentage of cells in S phase for Tet-On c-MYC–expressing Panc 10.05, HPAF-II, and PDX220 cells that were treated for 7 d with DMSO (Ctrl); 2 μg/ml Dox; 5–10 μM CQ and 2.5 μM palbociclib (P/CQ); or D/P/CQ. Control and Dox-treated sample percentages are continued from Fig. 3 B; experiments were performed at the same time as P/CQ- and D/P/CQ-treated samples (*n* = 3 from two independent experiments, mean ± SEM). *, P < 0.05; **, P < 0.01; ns, not significant by Student’s two-sided *t* test. **(E)** Immunoblot analysis of lysates generated from Tet-On c-MYC–expressing cells that were treated for 7 d with DMSO (Ctrl), 2 μg/ml Dox (D), 5–10 μM CQ, 2.5 μM palbociclib (P), or the indicated combinations (representative blots from two independent experiments). Lysates were blotted for c-MYC, pRB (phospho-S807/811), RB, CCNB1, CCNA2, and ACTB. Densitometry quantitation of c-MYC and pRB protein levels normalized to actin are shown. Source data are available for this figure: SourceData F5.

**Figure S3.**
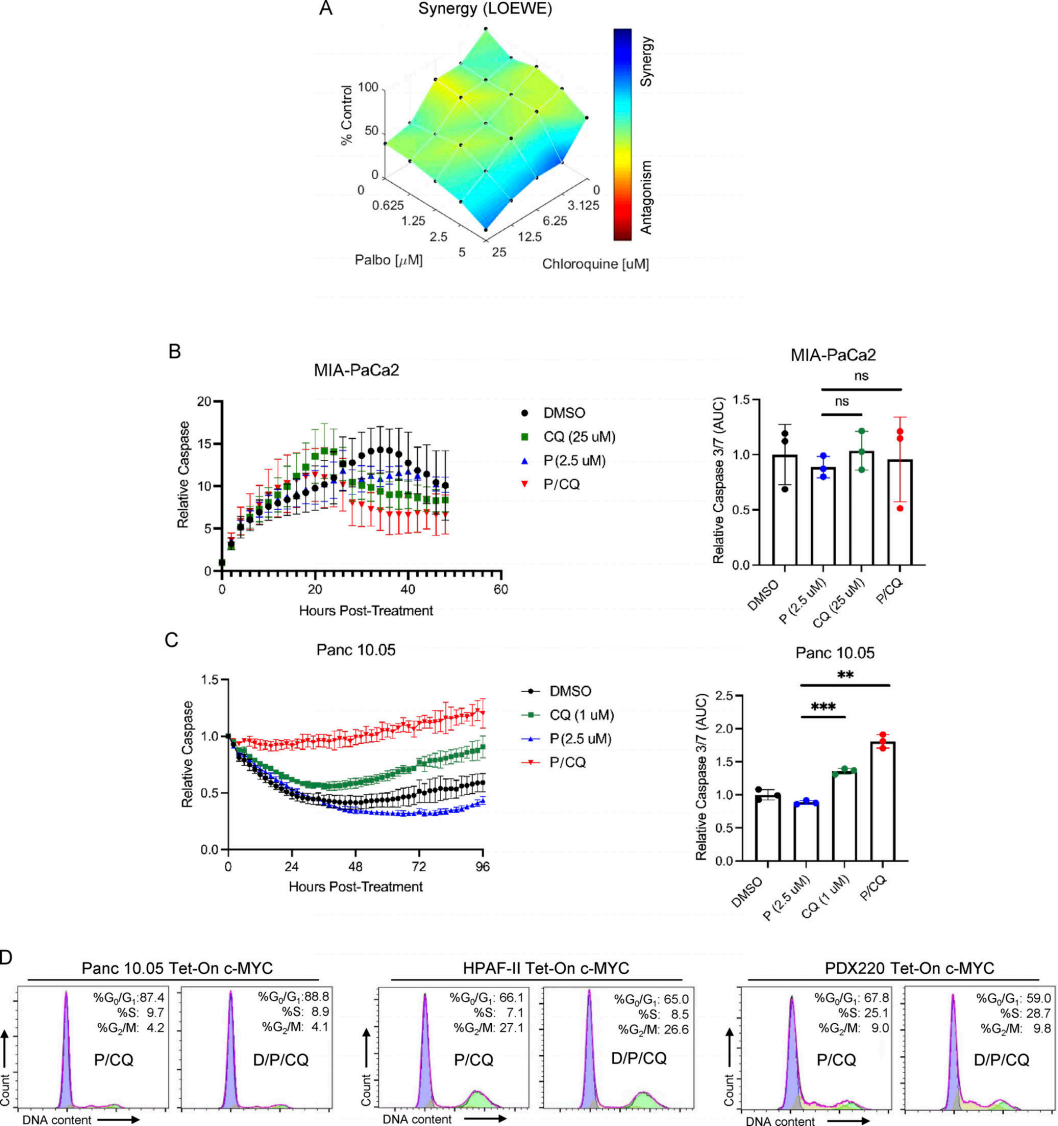
**Dual treatment with P/CQ prevents PDAC cell growth. (A) **MIA-PaCa2 cells were treated with either palbociclib or CQ alone or in combination at the indicated concentrations. Endpoint ATP was quantified, and Combenefit was used to calculate synergy with the Loewe model in an experiment that was performed twice (*n* = 2). **(B and C)** Cells were treated with palbociclib or CQ alone or in combination and caspase 3/7 activity was measured over time (left). The area under the curve was calculated at 48 h of treatment for MIA-PaCa2 or at 96 h for Panc 10.05, and mean values with error bars that represent SD are shown (right). A one-way ANOVA was utilized to determine statistical significance between the single agent controls and the combination group for an experiment that was performed once (*n* = 3); **, P < 0.01; ***, P < 0.001. **(D)** Representative cell cycle histograms with percentages of cells in G_0_/G_1_, S, and G_2_/M phases are shown for Panc 10.05, HPAF-II, and PDX220 cells expressing Tet-On c-MYC. Cells were treated for 7 d with palbociclib (P, 2.5–5 μM) and CQ (10–20 μM) with or without 2 μg/ml Dox (D).

To determine whether the combination of CDK4/6 plus lysosome inhibition prevented proliferation, we conducted in vitro growth assays using cells treated with (1) DMSO, (2) palbociclib, (3) CQ, or (4) P/CQ. Compared to controls, P/CQ demonstrated a significant synergistic anti-proliferative effect (Fig. 5 A and Fig. S3 A). Additionally, MIA-PaCa2 cells treated with CQ plus either ribociclib or abemaciclib again resulted in a significant anti-proliferative effect compared to controls (Fig. 5, B and C).

We observed that treatment of PDAC cells with trametinib increased the proportion of cells in G_0_/G_1_ phase; however, the addition of CQ had no further effect (Fig. S2, E and F). To determine whether the addition of CQ to palbociclib had any further effect on cell cycle progression, MIA-PaCa2, PDX220, and HPAF-II cells were treated with palbociclib or P/CQ (Fig. S2, E–G). Similar to what we observed for T/CQ, the addition of CQ to palbociclib did not increase the proportion of cells in G_0_/G_1_ phase.

To determine whether P/CQ treatment resulted in cell death, we assessed caspase 3/7 activity. Although there was no difference between the controls and combination groups in MIA-PaCa2 cells, we observed an increase in caspase 3/7 activity in P/CQ-treated Panc 10.05 cells (Fig. S3, B and C). Taken together, these data suggest that autophagy is a protective mechanism that facilitates PDAC cell growth in the face of CDK4/6 inhibition.

### Palbociclib plus CQ prevents c-MYC–mediated cell cycle progression

Given the ability of c-MYC to overcome the proliferative arrest induced by trametinib or T/CQ, we tested whether cells with elevated c-MYC expression are sensitive to CDK4/6 inhibition. As expected, palbociclib and P/CQ induced significant cell cycle arrest relative to control (Fig. S2, E–G). Indeed, the effects of palbociclib or P/CQ on S-phase progression were similar to those observed in response to trametinib or T/CQ (Fig. S2, E and F). Importantly, the ectopic expression of c-MYC^WT^ or c-MYC^T58A^ failed to overcome palbociclib or P/CQ-mediated cell cycle arrest (Fig. 5 D and Fig. S2, B–D, and F). Furthermore, while treatment with P/CQ and T/CQ elicited a similar effect on cell cycle regulator expression, ectopic expression of c-MYC^WT^ or c-MYC^T58A^ failed to promote RB phosphorylation or induce expression of cyclins A2 or B1 after P/CQ (Fig. 5 E and Fig. S2, H–L). Hence, these data demonstrate that c-MYC cannot overcome cell cycle arrest elicited by P/CQ.

### The combination of palbociclib plus CQ induces tumor stasis in PDAC with ectopically expressed c-MYC

**Figure 6.**
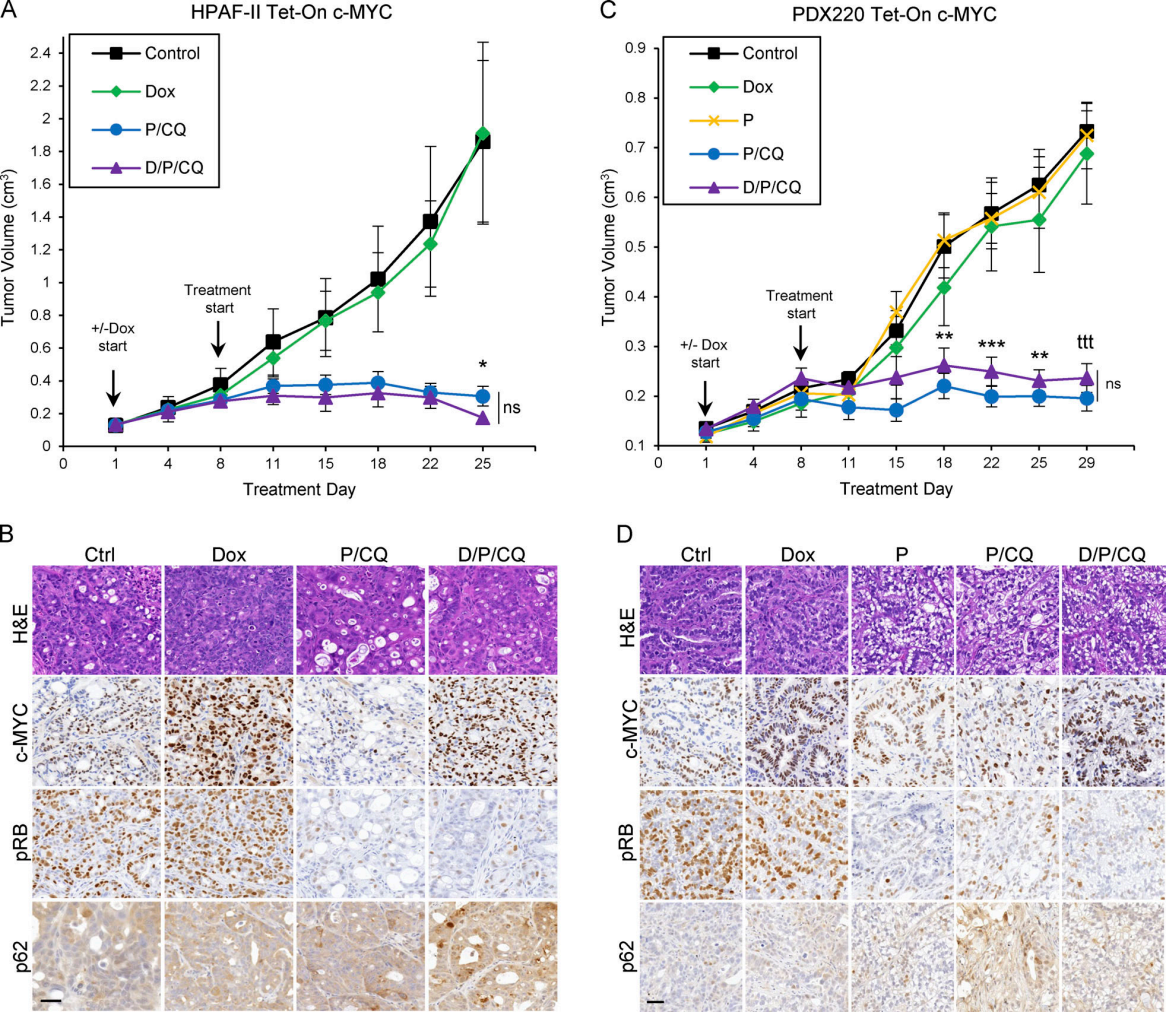
**P/CQ prevents growth of PDAC xenografts that express elevated c-MYC. (A and C)** The tumor volumes of xenografted Tet-On c-MYC–expressing HPAF-II and PDX220 cells are shown. Tumor-bearing mice were treated with: (1) corn oil (control, *n =* 5, *n =* 8, black lines); (2) 625 mg/kg Dox (*n =* 7, *n =* 8 green lines); (3) 100 mg/kg palbociclib (PDX220 only, P, *n =* 8, yellow line); (4) P/CQ (*n =* 4, *n =* 8, blue lines) or (5) D/P/CQ (*n =* 4, *n* = 8, purple lines), where *n* equals the number of mice for each cell type, respectively. Center values are the mean ± SEM. *, P < 0.05, **, P < 0.01; ***, P < 0.005; ^ttt^, P < 0.00005, Student’s two-sided *t* test. **(B and D)** Representative IHC sections of xenografted HPAF-II or PDX220 Tet-On c-MYC tumors from mice that were treated as in A and C. Sections were stained with H&E or with antibodies against c-MYC, pRB, or p62, as indicated. Scale bars are 50 μm.

**Figure S4.**
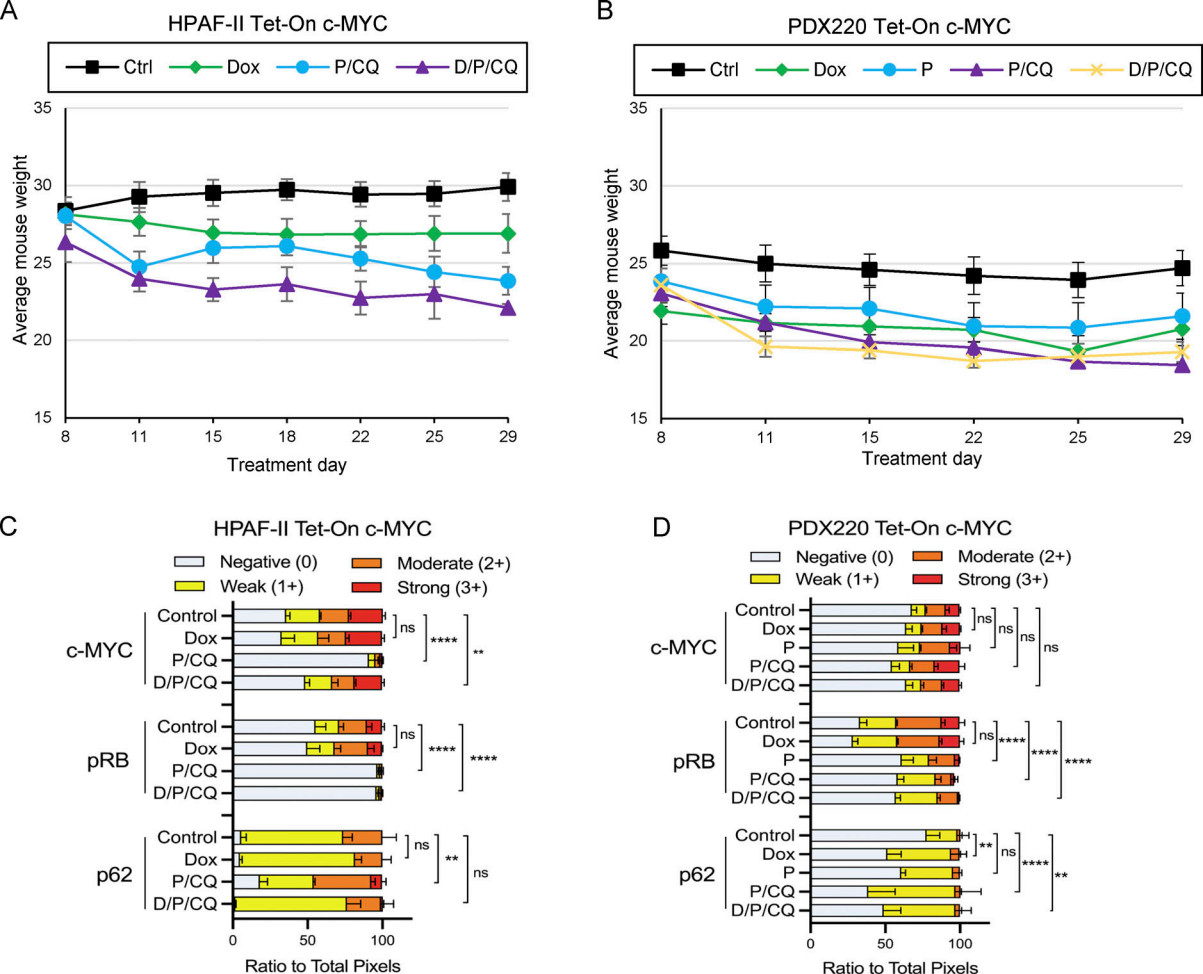
**P/CQ treatment is tolerated in mice. (A and B)** Average weights for mice bearing Tet-On c-MYC HPAF-II or PDX220 cells. Error bars represent SEM. **(C and D)** Quantitation of IHC performed on HPAF-II or PDX220 Tet-On c-MYC xenograft tumors shown in Fig. 6. Two-way ANOVA was used to determine significance. **, P < 0.005; ****, P < 0.0005.

To determine whether P/CQ treatment prevents PDAC tumor growth with or without the expression of c-MYC, mice were xenografted with Tet-On c-MYC–expressing HPAF-II or PDX220 cells (Fig. 6, A and C) and treated with (1) vehicle (control), (2) Dox to induce c-MYC expression (Dox), (3) palbociclib (P, only in PDX220), (4) P/CQ, or (5) Dox plus P/CQ (D/P/CQ). P/CQ treatment arrested tumorigenic growth regardless of c-MYC expression. IHC analysis confirmed elevated c-MYC expression in tumors growing in Dox-treated mice and reduced phosphorylation of RB with palbociclib treatment (Fig. 6, B and D; and Fig. S4, C and D). These results demonstrate that the treatment of xenografted PDAC tumors with P/CQ inhibited growth in a c-MYC–independent manner.

### Patient 1 displayed a CA 19-9 tumor marker reduction in response to treatment with palbociclib plus HCQ

**Figure 7.**
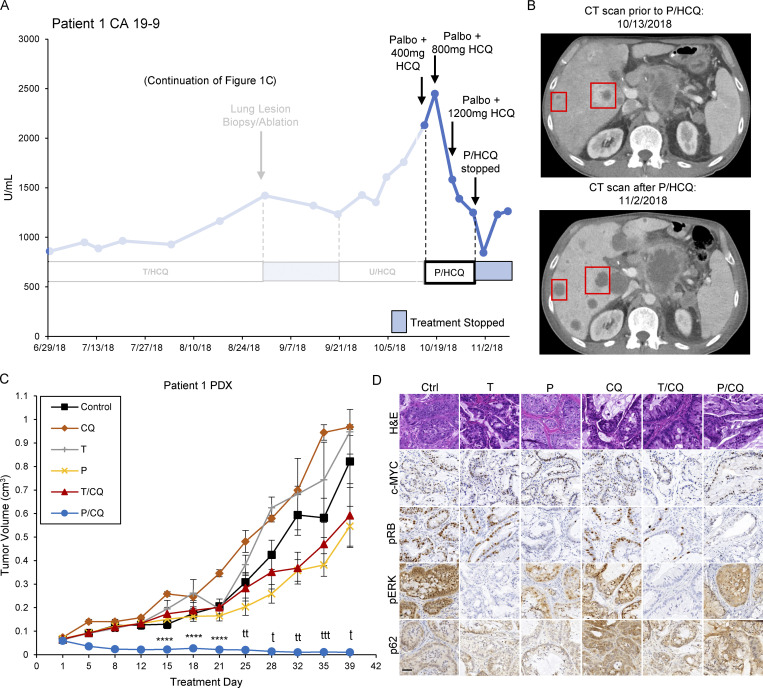
**Treatment of Patient 1 with P/HCQ results in reductions in CA19-9 and overall tumor burden. (A)** Patient 1 CA19-9 tumor marker levels (continued from Fig. 1 C), are displayed and annotated with dates and treatments administered throughout the clinical course. CA19-9 levels dropped after 125 mg palbociclib (q.d.) plus 800 mg (400 mg b.i.d.) HCQ and rebounded after P/HCQ cessation. U/HCQ, ulixertinib and HCQ. **(B)** Patient 1 CT scans before (top) and after (bottom) P/HCQ treatment. Red boxes indicate liver metastases and areas with darker shading indicate liquefactive necrosis. **(C)** Patient 1 PDX tumor volumes are shown. Tumor-bearing mice were treated as follows: (1) corn oil (control, *n* = 6, black line); (2) 50 mg/kg CQ (*n* = 6, orange line); (3) 1 mg/kg trametinib (T, *n* = 6, gray line); (4) 100 mg/kg palbociclib (P, *n* = 6, yellow line); (5) T/CQ (*n* = 6, red line); or (6)P/CQ (*n* = 6, blue line), where *n* equals the number of mice. Center values are the mean ± SEM. ****, P < 0.001; ^t^, P < 0.0005; ^tt^, P < 0.0001; ^ttt^, P < 0.00005 by two-sided Student’s *t* test. **(D)** Representative IHC images of Patient 1 PDX tumors from mice that were treated as in C. Sections were stained with H&E (top row) or with antibodies against c-MYC, pRB, pERK1/2, or p62, as indicated. Scale bar is 50 μm.

**Figure S5.**
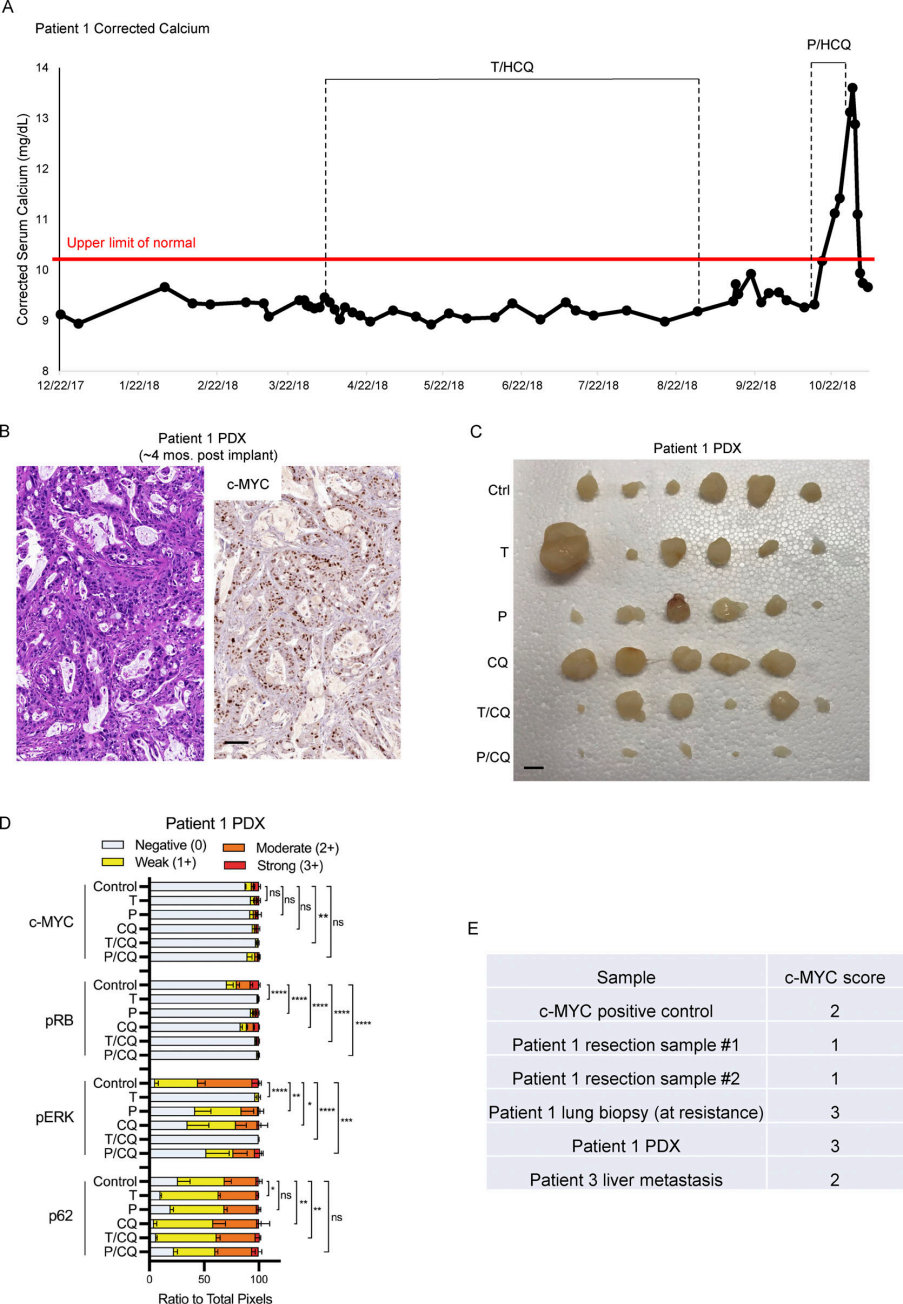
**Patient 1 PDX retains elevated c-MYC expression and is sensitive to P/CQ co-treatment. (A)** Patient 1 calcium concentrations were monitored over the course of treatment. Annotations describe duration of T/HCQ and P/HCQ treatment. **(B)** Patient 1 PDX was xenografted and tumors were analyzed by H&E staining and c-MYC IHC. **(C)** Tumors excised from mice treated in Fig. 7 are shown. Patient 1 PDX tumor chunks were xenografted into NOD/SCID mice and mice were treated with (1) corn oil (Ctrl), (2) 1 mg/kg trametinib (T), (3) 100 mg/kg palbociclib (P), (4) 50 mg/kg CQ, (5) T/CQ, or P/CQ. Bar represents 1 cm. **(D)** Quantitation of IHC performed on Patient 1 PDX xenografts shown in Fig. 7. *, P < 0.05; **, P < 0.005; ***, P < 0.0005; ****, P < 0.0001; and ns, not significant by two-way ANOVA. **(E)** IHC samples obtained from Patient 1 resection, Patient 1–derived xenograft (4 mo after implant), Patient 1 lung biopsy (at resistance), and Patient 3 (liver metastasis) were scored for c-MYC. Scoring definitions are as follows: 0 = extremely weak to no staining; 1 = <10%; 2 = 10–50%; 3 = >50% (Schleger et al., 2002).

After 177 d of T_2_/HCQ_1200_ treatment, Patient 1 displayed the signs of disease progression including increased CA19-9 and tumor size on CT imaging (Fig. 7 A). Patient 1 was switched to a combination of an ERK1/2 inhibitor (ulixertinib) plus HCQ based on the apparent sensitivity of a BRAF-driven PDAC to ulixertinib after the emergence of resistance to trametinib (Aguirre et al., 2018). However, the patient failed to respond. Subsequent analysis of a lung metastasis revealed increased copy number on chromosome 8p including *c-MYC* compared to the primary tumor. Based on our preclinical data, the patient consented to be treated with palbocilclib (125 mg every day [q.d.]) plus HCQ (800 mg, 400 mg twice daily [b.i.d.] with escalation to 1,200 mg, 600 mg b.i.d., P/HCQ) therapy on an off-label/off-trial basis. CA19-9 levels decreased after 9 d of treatment and continued to do so for 10 d, suggesting a response to P/HCQ (Fig. 7 A). Moreover, 3 wk after initiation of P/HCQ treatment, CT imaging suggested liquefactive necrosis of metastatic liver lesions (Fig. 7 B). Additionally, the patient developed hypercalcemia of malignancy (HHM) and testing revealed an elevated parathyroid hormone-related peptide (PTHrP) of 106.0 pmol/liter (normal range 0–2.3 pmol/liter) suggesting tumor cell lysis (Fig. S5 A). The patient’s HHM was successfully treated. Sadly however, the patient failed to thrive and discontinued P/HCQ treatment, at which time his serum CA19-9 level increased and the patient opted to go on hospice.

Subsequently, we established a PDX model from the biopsied lung lesion in NOD/SCID mice. IHC analysis revealed the expression of c-MYC similar to the biopsied lung lesion (Fig. S5 B). To determine whether Patient 1’s response to P/HCQ was due to the effect of any single agent, mice bearing the T/HCQ-resistant tumor were treated with (1) vehicle, (2) CQ, (3) trametinib, (4) palbociclib, (5) T/CQ, or (6) P/CQ. Patient 1’s PDX tumor was resistant to all treatments except P/CQ (Fig. 7 C and Fig. S5 C), further suggesting that P/CQ treatment is effective against T/HCQ-resistant PDAC with elevated c-MYC expression. IHC analysis of tumors from vehicle vs. drug-treated mice confirmed (1) c-MYC expression, (2) decreased pERK1/2 in trametinib-treated mice, (3) decreased pRB in trametinib- and palbociclib-treated mice, and (4) elevated p62^SQSTM1^ in CQ-treated mice (Fig. 7 D and Fig. S5 D). Taken together, these preclinical and clinical findings suggest that the combination of palbociclib plus HCQ may be effective for treating PDAC with c-MYC–mediated resistance to trametinib and HCQ.

## Discussion

The combination of T/HCQ may hold promise as a treatment for PDAC; however, we have identified cases of intrinsic and acquired resistance in PDAC patients. Here, we reported the role of c-MYC as both a potential biomarker and a mediator of resistance to combined inhibition of KRAS>RAF>MEK>ERK signaling, with trametinib, plus autophagy, with HCQ, in preclinical models and patients. Since c-MYC is frequently amplified in PDAC (Mahlamaki et al., 2002; Schleger et al., 2002), it will be critical to verify whether c-MYC is a bona fide biomarker of T/HCQ resistance through examination of additional patient samples and natural histories obtained from clinical trials. Copy number alterations do not account for all cases of c-MYC over-expression and discordances exist between *c-MYC* copy number gains and c-MYC protein expression in PDAC (Schleger et al., 2002), which is expected as the regulatory mechanisms that control c-MYC expression are numerous and complex (Hessmann et al., 2016). Analysis of DNA from Patient 3’s tumor did not reveal a copy number alteration of *c-MYC*, yet we observed elevated c-MYC expression in a liver biopsy taken prior to the observation of intrinsic T/HCQ resistance. Indeed, it is also clear that c-MYC is regulated by the KRAS>RAF>MEK>ERK pathway in some PDAC cell lines, which may contribute to sensitivity or resistance to T/HCQ therapy (Gysin et al., 2012; Hayes et al., 2016; Vaseva et al., 2018). Finally, non-tumor cell autonomous mechanisms within the PDAC microenvironment might also influence c-MYC expression as hyperglycemia, acidic fibroblast growth factor from cancer-associated fibroblasts, and IL4 or IL13 from tumor-infiltrating Th2 cells have all been reported to influence c-MYC expression in PDAC cells (Sato et al., 2020; Bhattacharyya et al., 2020; Dey et al., 2020).

Observations reported here suggest that Clinical Laboratory Improvement Amendments (CLIA)–certified c-MYC analysis and scoring of tumor biopsies may be necessary to accurately determine relative c-MYC expression levels, rather than analysis of genomic DNA or RNA alone. Although our patient samples were assessed for c-MYC expression by IHC performed by a CLIA-certified laboratory (Fig. S5 E) and blindly scored by a board-certified pathologist (Schleger et al., 2002), caution must be used when drawing conclusions from a small sample size. However, the data presented here makes a strong connection between elevated expression of c-MYC and resistance to T/HCQ therapy.

c-MYC is one of the original cooperating partners with activated RAS in oncogenic transformation assays (Land et al., 1983). We and others observed reduced c-MYC expression following inhibition of MAPK signaling in the majority of human PDAC cell lines tested (Gysin et al., 2012; Hayes et al., 2016; Vaseva et al., 2018). In addition to the effects on c-MYC transcription, ERK1/2 activity also regulates c-MYC stability by phosphorylating serine 62 that, in turn, regulates proteasomal destruction of c-MYC (Bahram et al., 2000; Sears et al., 2000). Previous work demonstrated that inhibition of MAPK signaling inhibits the PDAC cell cycle and implicated that c-MYC suppression may be responsible for the G_1_ cell cycle arrest (Gysin et al., 2012). Here, we specifically demonstrate that conditional expression of c-MYC overcomes MEK1/2 inhibitor-mediated cell cycle arrest, which was likely mediated by alterations in cyclins, CDKs, and CKIs. It was perhaps surprising that c-MYC did not drive elevated expression of either D- or E-type cyclins (Bouchard et al., 1999). However, given the importance of p27^KIP1^ in enforcing MEK1/2 inhibitor–mediated G_1_ cell cycle arrest, it is likely that the c-MYC–mediated increased expression of cyclins A2 and B1 and decreased p27^KIP1^ expression was sufficient to overcome the cell cycle arrest observed after T/CQ treatment (Gysin et al., 2005; Qi et al., 2007). However, given that c-MYC is a highly pleiotropic transcription factor implicated in the regulation of the expression of ∼15% of all human genes (Dang et al., 2006), it is challenging to pinpoint a single mechanism by which it promotes resistance to T/HCQ treatment. Recently, MEK1/2 inhibitor–induced downregulation of c-MYC expression was shown to allow for increased occupancy of MiT/TFE transcription factors at CLEAR (coordinated lysosomal expression and regulation) elements within the promoters of numerous lysosome genes in PDAC, which resulted in elevated lysosome biogenesis and ultimately increased ferritinophagy required for PDAC homeostasis (Ravichandran et al., 2022). Whether elevated or MAPK inhibitor–resistant c-MYC expression impacts trametinib-induced autophagy or lysosome activity is the focus of ongoing work. If c-MYC expression remains stable after MAPK inhibitor treatment, it would be expected to compete with MiT/TFE binding to CLEAR elements and thereby blunt cytoprotective autophagy by reducing lysosome gene expression, and potentially increase sensitivity to single-agent trametinib. However, Patient 1 PDX xenografts were insensitive to single-agent trametinib (Fig. 7 C). Additionally, PDAC cells with trametinib-resistant c-MYC expression are less sensitive to single-agent MEK1/2 inhibition, exhibiting exponentially higher IC50 values, as a result of MYC-driven metabolic reprogramming to maintain nucleotide biosynthesis (Santana-Codina et al., 2018). These data suggest that c-MYC–mediated resistance to T/HCQ may not only act solely through the downregulation of autophagy, but also through uncoupling the dependence of PDAC on trametinib-induced cytoprotective autophagic flux by way of c-MYC–mediated regulation of metabolic processes such as nucleotide biosynthesis. Thus, while sustained c-MYC expression may reduce trametinib-induced autophagy through the transcriptional downregulation of CLEAR-regulated genes, it may simultaneously upregulate biosynthetic pathways that shift PDAC away from reliance upon cytoprotective autophagy, concomitantly reducing the cytotoxic effect of lysosome inhibition with HCQ. These questions warrant studies to identify additional MYC-mediated resistance mechanisms that can be targeted for therapeutic benefit.

We noted that reducing c-MYC expression conferred sensitivity to tumors that had grown through T/CQ treatment and allowed for T/CQ-mediated tumor regression. We observed decreasing c-MYC expression with alisertib, and also in tumors co-treated with alisertib plus T/CQ. The combination of alisertib plus T/CQ resulted in tumor regression, suggesting that reducing c-MYC expression confers sensitivity. However, reducing c-MYC expression alone induces tumor regression in mouse models of PDAC (Vaseva et al., 2018; Tsang et al., 2017); therefore, determining whether reducing c-MYC expression confers sensitivity to T/CQ is technically challenging. Indeed, we utilized Dox-regulated shRNAs to inhibit c-MYC expression in PANC-1 cells and demonstrated a near complete inhibition of cell growth in vitro; therefore, demonstrating susceptibility to T/CQ in vivo with reduced c-MYC expression through shRNA-mediated knock down was not feasible.

In addition to high-frequency alteration of *KRAS*, *TP53*, and *SMAD4*, ∼30% of PDACs harbor inactivating mutations in *CDKN2A*, which encodes for two tumor suppressors: (1) *INK4A* that encodes p16^INK4A^, a stoichiometric inhibitor of CDK4/6; and (2) *ARF* that encodes p14^ARF^, a regulator of the MDM2>TP53 pathway (Otto and Sicinski, 2017). However, despite the fact that PDACs are driven by gene mutations that result in hyperactive D-type cyclins and CDK4/6, PDACs are noted for resistance to pharmacological inhibitors of CDK4/6 (Kumarasamy et al., 2020). Indeed, a Phase II clinical trial demonstrated that palbociclib had no clinical efficacy in PDAC patients harboring tumors with inactivated *CDKN2A* (Baghdadi et al., 2019). Although we observed that treatment of cultured PDAC cells with palbociclib reduced cell proliferation, we confirmed palbociclib’s lack of single-agent anti-tumor activity in vivo. However, as with inhibition of KRAS>RAF>MEK>ERK signaling, treatment of PDAC cells with CDK4/6 inhibition increased autophagy. Independently, and consistent with our findings, another group recently reported increased autophagy in palbociclib-treated PANC-1 cells (Ji et al., 2020). Furthermore, we observed that CQ-sensitized cultured PDAC cells to palbociclib treatment and resulted in stasis of xenografted tumors. Additionally, PDAC tumors resistant to T/CQ due to elevated c-MYC remained sensitive to treatment with P/CQ. Although it is tempting to speculate that the ability of palbociclib to induce autophagy is due to the inhibitory effects on the cell cycle, CDK4/6 are reported to phosphorylate non-canonical proteins involved in nutrient sensing including LKB1, AMPK, and mTORC1, known upstream regulators of ULK1-mediated autophagic degradation (Franco et al., 2016; Casimiro et al., 2017; Martínez-Carreres et al., 2019; Kim et al., 2011; Romero-Pozuelo et al., 2020). Additionally, CDK4/6 have also been reported to phosphorylate rate-limiting metabolic enzymes including phosphofructokinase and pyruvate kinase, which dictate flux through the biosynthetic pentose phosphate pathway and serine biosynthesis pathways, respectively, both of which are supported by autophagy (Wang et al., 2017; Guo et al., 2016). Hence, future work should focus on the signaling mechanisms that link CDK4/6-CyclinD activity to nutrient sensing and autophagy independent of their role in cell cycle regulation.

Finally, we noted that Patient 1 developed HHM because of elevated serum PTHrP, a tumor-secreted protein that stimulates calcium resorption. HHM is not a noted toxicity of either palbociclib or HCQ treatment. Moreover, although some PDACs express PTHrP, most reported HHM cases in pancreatic cancer are found in patients with neuroendocrine tumors (Miraliakbari et al., 1992; Bouvet et al., 2001). However, it is possible that elevated expression of c-MYC may have promoted an epithelial-to-neuroendocrine transition, as has been noted in other malignancies (Farrell et al., 2017). Although P/HCQ treatment elicited cytostatic effects in a preclinical model, it is possible that P/HCQ may be a useful combination strategy in late-stage PDAC patients regardless of c-MYC expression. Hence, it will be important to evaluate whether markers of resistance to palbociclib treatment, such as RB inactivation or CDK2 amplification, predict resistance to P/HCQ in PDAC (Kumarasamy et al., 2020). Hence, in summary, P/HCQ treatment may represent another possible therapeutic modality for PDAC patients and warrants further exploration in a rigorous clinical trial.

## Materials and methods

### Study design

The overall research objective was to determine whether P/CQ overcomes c-MYC–mediated resistance to T/CQ in PDAC. After observing the correlation between c-MYC expression and T/CQ resistance, the first objective was to determine whether c-MYC alone promotes T/HCQ resistance. The second objective was to identify how c-MYC mediates resistance and whether P/CQ overcomes T/CQ resistance. To accomplish these objectives, the following methods were used: xenograft mouse studies, flow cytometry, proliferation assays, IHC staining, histopathology analysis, immunoblotting, electron microscopy, and fluorescence microscopy. The precise values for replicating numbers for each experiment are detailed in figure legends. The sample size was calculated based on prior knowledge and literature. Experiments were not blinded. Mice were randomized to groups before the start of treatment, assessed for indications of toxicity, and euthanized at a predefined humane endpoint according to the University of Utah’s Institutional Animal Care and Use Committee protocol. No data were excluded.

### Patient samples

Human tissue sample collection was performed after written informed patient consent was obtained under the Molecular Classifications of Cancer protocol (IRB#10924) and the Huntsman Cancer Institute (HCI) Total Cancer protocol (IRB#89989).

### Cells lines

All cell lines were cultured in 5% (vol/vol) CO2 at 37°C in medium containing 1% penicillin/streptomycin. HPAF-II, Panc 10.05, PANC-1, and MIA PaCa-2 cell lines were obtained from ATCC. HPAF-II was cultured in EMEM (ATCC) with 10% (vol/vol) FBS. Panc 10.05 was cultured in RPMI-1640 (Gibco) supplemented with 10 U/ml human recombinant insulin (Sigma-Aldrich) and 10% (vol/vol) FBS. The PDX220 cell line was derived from a PDAC patient as described previously (Kinsey et al., 2019) and maintained in 1:1 DMEM/F-12 (Gibco) with 10% (vol/vol) FBS, as were PANC-1 and MIA PaCa-2 cells. Cell lines were tested for *Mycoplasma* contamination regularly by PCR and discarded if positive.

### Lentiviral transduction

pUltra-Auto (described in Kinsey et al., 2019) and pLVX-TetOne-Puro c-MYC^T58A^ (a gift from Donald Ayer; HCI, Salt Lake City, UT, USA) lentiviral constructs were used to express mCherry-eGFP-LC3 and Tet-On c-MYC in cell lines, respectively. pLVX-TetOne-Puro c-MYC^T58A^ was mutated to wild-type c-MYC using the QuikChange II XL Site-Directed Mutagenesis Kit (Agilent). Lentivirus production was performed as described previously (Kinsey et al., 2019). pUltra auto-transduced cells were selected through FACS for mCherry expression while pLVX-TetOne c-MYC wild-type or T58A-transduced cells were selected with 2 μg/ml puromycin.

### Autophagic flux assays

Cell lines expressing the AFR, generated as described previously (Kinsey et al., 2019), were treated as indicated for 48 h with the exception of PDX220 cells, which were treated for 72 h. Cells were detached with 0.25% (vol/vol) Trypsin-EDTA (Gibco) and resuspended in media containing 1 μM SYTOX Blue stain. A CytoFLEX (Beckman Coulter) flow cytometer was utilized to analyze eGFP and mCherry fluorescence. Gates were set to exclude dead cells and doublets in CytExpert software. DMSO controls were gated into thirds (high, intermediate, and low autophagy) and set gates were maintained throughout individual experiments. FlowJo was used to calculate the ratio of mCherry to eGFP. All experiments were conducted in biological triplicate. A two-tailed *t*-test was used to calculate significant differences between the percentage of cells in the high autophagy (red) gate of the DMSO control and experimental groups.

### In vitro proliferation assays

Cells were seeded at 7,000–10,000 cells/well in 96-well plates, treated, and imaged using the IncuCyte Live-Cell Analysis System. Data were collected and analyzed with the Incucyte ZOOM 2016B software. Cell line–specific masks were generated to measure confluence over time. One-way ANOVA was used to determine the statistical significance between single-agent treatment groups and the combination group.

### Cell cycle analysis

Cells were plated on 10 cm^2^ dishes and treated for 7 d. 0.25% (vol/vol) trypsin-EDTA (Gibco) was used to harvest cells, which were washed twice with PBS and fixed with ice-cold 70% (vol/vol) ethanol at −20°C for at least 24 h. Fixed cells were washed with PBS twice and incubated in 0.1% (vol/vol) Triton X-100 (Sigma-Aldrich) and 1 μg/ml DAPI (Thermo Fisher Scientific) in PBS for 30 min. At least 30,000 single cells were analyzed on a CytoFLEX (Beckman Coulter) flow cytometer. Cell cycle phase determination was performed in FlowJo using Dean-Jett-Fox modeling (Fox, 1980).

### Immunoblotting

Lysates were prepared by direct solubilization in sample buffer: fresh 2× sample buffer (Thermo Fisher Scientific) was added to cells, sonicated for 5 s, and boiled for 10 min. The equal volumes of lysates were separated on NuPage 4–12% (wt/vol) Bis-Tris gels (Thermo Fisher Scientific) and transferred to a polyvinylidene fluoride membrane (Invitrogen) using the iBLOT 2 Dry Blotting system (Thermo Fisher Scientific). Membranes were blocked with Odyssey Blocking Buffer (LI-COR) for 1 h at room temperature and incubated with primary antibodies overnight at 4°C. Antibodies used are as follows (used at 1:1,000 unless noted otherwise): 1:2,000 phospho-T202/Y204 p44/42 MAPK (ERK1/2; CST:4370), total ERK1/2 (CST:4696); p-RB 807/811 1:1,000 (CST 8516S); RB (CST:9309S); c-MYC (CST:5605S); E2F1 (CST:3742S); Cyclin B1 (CST:12231S); Cyclin A2 (CST:91500S); Cyclin D2 (CST:3741S); Cyclin E1 (CST:20808S); CDK2 (CST:2546S); CDK4 (CST:12790S); CDK6 (CST:3136S); p27^KIP1^, (CST:3686S); p21^WAF1/CIP1^ (CST:2947S); 1:1,000 Vinculin (CST:13901); 1:2,000 GAPDH (CST:97166S); 1:5,000 β-actin (CST:4970S). Standard immunoblotting procedures using Alexa 680– or 800–conjugated species-specific secondary antibodies were followed and images were acquired with a LI-COR CLx digital fluorescence scanner. Protein bands from acquired LI-COR images were quantified using the Image Studio software.

### Mice

NOD/SCID mice were bred and maintained in a pathogen-free facility by HCI’s Preclinical Research Resource. All animal experiments were performed in accordance with protocols approved by the University of Utah Institutional Animal Care and Use Committee, and we have complied with all relevant ethical regulations.

### Xenograft assays

Xenografted tumors were established by subcutaneous injection of 2 × 10^6^ HPAF-II, PDX220 or PANC-1 cells, resuspended in 50 μl Matrigel (Corning Life Sciences), into NOD/SCID mice. Patient 1 tumor xenografts were implanted subcutaneously by HCI’s Preclinical Research Resource. Mice with xenografted tumors were administered drugs through oral gavage (p.o.) daily as follows: (1) vehicle control (corn oil); (2) trametinib at 1 mg/kg (in corn oil); (3) CQ at 50 mg/kg (in corn oil); (4) combination of trametinib plus CQ; (5) alisertib at 20 mg/kg (in 10% DMSO, 90% corn oil); (6) combination of alisertib, trametinib, plus CQ; (7) palbociclib 100 mg/kg (in 50 mM sodium lactate, pH 4.0); or (8) combination of palbociclib plus CQ. For mice with tumors expressing Tet-On c-MYC, mice were fed either standard chow or chow containing 625 mg/kg Dox (Envigo Teklad). Tumors were measured twice weekly using calipers and tumor volume (in cm^3^) was calculated with the formula: (4/3) * ∏ * [(length + width)/2]^3^. Significant differences in tumor size were calculated using a two-tailed *t *test.

### PDX assays

Tumor tissue was obtained after written informed consent was obtained from the patient, according to a tissue collection protocol (University of Utah IRB 10924) approved by the HCI Institutional Review Board. Tumor specimens were implanted subcutaneously into NOD/SCID mice. Patient 1 PDX was derived from a lung metastasis that demonstrated stability during treatment, then grew progressively in a 68-yr-old man who had been previously treated with standard-of-care therapies, as described previously (Kinsey et al., 2019). For serial propagation and experiments, 50–70 mg tumor fragments were implanted bilaterally into the flanks of NOD/SCID mice. When tumors were measurable, treatment was initiated as described above.

### IHC

Tumors were fixed for 24 h in 10% (vol/vol) formalin, transferred to 70% (vol/vol) ethanol, embedded in paraffin, and 4-micron thick sections were cut. Deparaffinized sections were treated with Bloxall (Vector Labs) followed by horse serum (Vector Labs). Primary antibodies were used to detect phospho-ERK (CST:D13.14.4E) 1:600; p62^SQSTM1^ (Progen:GP62-C) 1:20; and pRB (CST:D20B12) 1:400, followed by anti-rabbit (Vector Labs) or anti–guinea pig (Vectastain) HRP-polymer. Primary antibody was visualized with DAB (Vector Labs), and counterstaining was performed with hematoxylin. IHC for c-MYC expression in xenografted human PDAC tumors was performed by ARUP Laboratories. Representative IHC images were obtained using a Pannoramic MIDI 3D Histech slide scanner (Epredia). IHC quantitation was performed using the 3DHistech scanner CaseViewer (version 2.4) at 20×. Representative IHC fields were quantified with the DensitoQuant algorithm. Staining intensities were classified as negative (0) or weak (1+), moderate (2+), and strong (3+) positive pixels, relative to total pixel number per field using default threshold ranges. Bars represent mean staining ratios for at least three fields ± SEM. The sum of weak, moderate, and strong averages (for c-MYC, pERK, and pRB) or moderate and strong (for p62) were used to determine P values (two-way ANOVA), which are listed in figure legends.

### In vitro Incucyte Caspase 3/7 assays

Cells were seeded at 10,000 cells/well in 96-well plates and cultured overnight. Cells were treated in triplicate in 20% (vol/vol) medium in EBSS with 5 nM Incucyte Caspase-3/7 Green Apoptosis Assay Reagent (Essen Bioscience). Cells were imaged with the Incucyte Live-Cell Analysis System, and GFP-positive cells were estimated over time. Prism 9 was used to calculate area under the curve. One-way ANOVA was used to determine statistical significance between single-agent treatment groups and the combination group.

### In vitro synergy assays

Cells were seeded in quadruplicate at 7,000 cells/well into a 96-well plate, cultured overnight, and then treated with palbociclib, CQ, or the combination at the indicated concentrations in 20% (vol/vol) medium in EBSS for 48 hours. At endpoint, cells were transferred to a white Nunc Microplate 96-well optical bottom plate and ATP was quantified using ATPlite 1step (Perkin Elmer). Luminescence was measured with a BioTek Synergy HTX plate reader. Data were normalized to an untreated control and analyzed with Combenefit software (Loewe model; Di Veroli et al., 2016).

### Electron microscopy

The cells were fixed with 2.5% glutaraldehyde and 1% paraformaldehyde in cacodylate buffer overnight at 4°C. Cells were post-fixed with 2% osmium tetroxide for 1 h at room temperature, washed with cacodylate buffer, and stained en bloc with aqueous saturated uranyl acetate. Two rinses were performed, and the cells were dehydrated in graded ethanol series (30%, 50%, 70%, 95% 2 × 100% 3 ×) followed by acetone (3×) and infiltrated and embedded in epon-araldite resin. Ultrathin sections at 70 nm thickness were obtained using Leica UC6 Ultratome and were collected on 200-mesh copper grids. Sections were counterstained by incubation with aqueous saturated UA for 10 min followed by staining with lead citrate for 5 min. Sections were viewed on a JEOL (JEM-1400 Plus) transmission electron microscope, operated at 120 kV, and images were acquired on a Gatan 2 K × 2 K digital camera. ImageJ software (National Institutes of Health) was used to quantify vesicle number and size.

### Acidic organelle quantification

PDAC cells were seeded at 30,000 cells/well into a 12-well plate in DMEM/F12, cultured overnight, and treated in biological triplicate or quadruplicate with drug. After 48 h, cells were treated with 100 nM LysoTracker Red DND-99 (Thermo Fisher Scientific) for 30 min and two drops of NucBlue Live Cell Stain Ready Probes reagent for 15 min. Images were captured with the EVOS FL Cell Imaging System (Thermo Fisher Scientific). ImageJ software (National Institutes of Health) was used to subtract background fluorescence and merge images. The signal intensity was quantified and divided by the total number of cells per field, which was counted using the cell counter plugin to quantify nuclei. This value was multiplied by 100 and graphed in Prism 9.

### Quantitation and statistical analysis

All data are expressed as mean with standard deviation or standard error bars as indicated in figure legends. The number of independent experiments is indicated by *n*. Statistical analyses were performed in GraphPad Prism 9 or Excel. P values were considered statistically significant if <0.05. Statistical tests used for each experiment are detailed above and in figure legends.

### Online supplemental material

Fig. S1 shows trametinib-induced reductions in c-MYC expression in human PDAC lines; c-MYC^T58A^–mediated resistance to T/CQ in xenografted mice; mouse weights for each treatment group; and changes in c-MYC expression with alisertib and/or trametinib treatment. Fig. S2 shows that c-MYC^WT^ or c-MYC^T58A^ expression drives cell cycle progression in T/CQ- but not P/CQ-treated human PDAC cell lines, including supplemental c-MYC^T58A^ cell cycle analysis, single-agent cell cycle analysis and immunoblots of cell cycle regulators. Fig. S3 shows synergy, apoptosis assessment, and representative cell cycle histograms for P/CQ- and D/P/CQ-treated PDAC cell lines. Fig. S4 shows mouse weights and IHC quantifications for treatment groups presented in Fig. 6. Fig. S5 shows Patient 1 PDX retains elevated c-MYC expression and is sensitive to P/CQ. Quantitation of IHC performed on Patient 1 PDX xenografts shown in Fig. 7 and scoring of IHC samples for c-MYC are also shown.

## Supplementary Material

SourceData F2 contains original blots for Fig. 2.

SourceData F3 contains original blots for Fig. 3.

SourceData F5 contains original blots for Fig. 5.

SourceData FS1 contains original blots for Fig. S1.

SourceData FS2 contains original blots for Fig. S2.

## Data Availability

All data are available in the main text or the supplemental materials.
