# Supplementary figures and images for: MYC-mediated resistance to trametinib and HCQ in PDAC is overcome by CDK4/6 and lysosomal inhibition
Source: J Exp Med. 2023 Jan 31;220(3):e20221524. doi: 10.1084/jem.20221524 (PMC9930170; doi:10.1084/jem.20221524)

F2A

HPAF-II Tet-On c-MYC

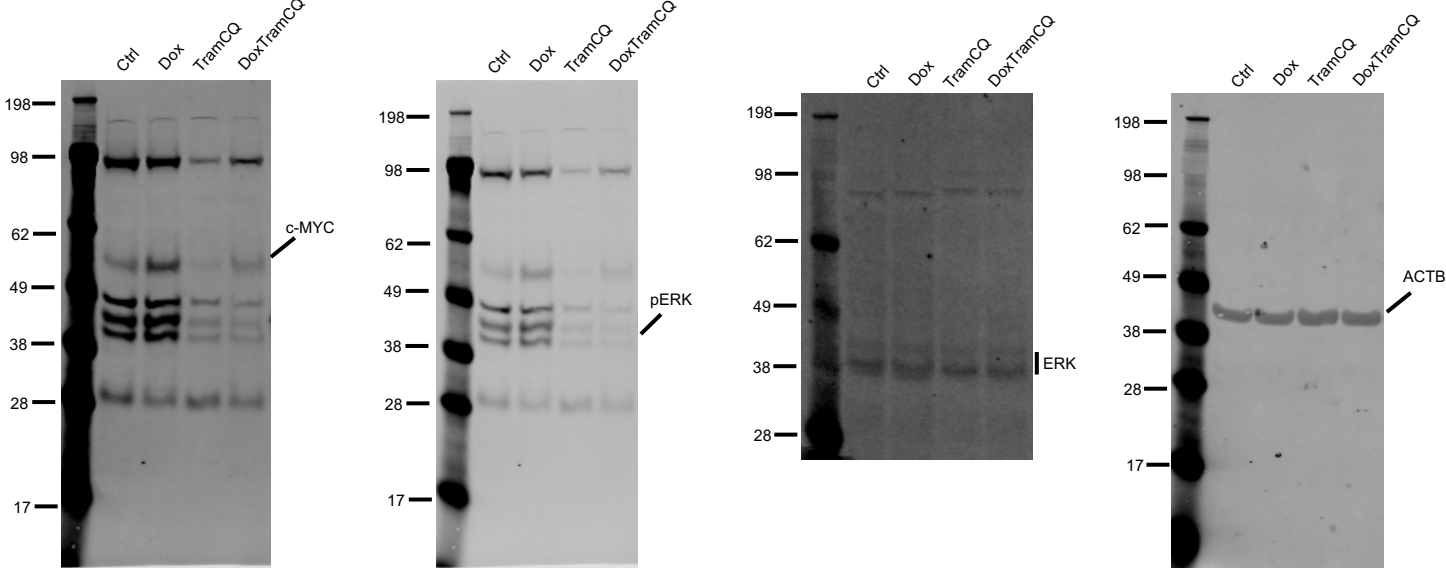

F2C

PDX220 Tet-On c-MYC

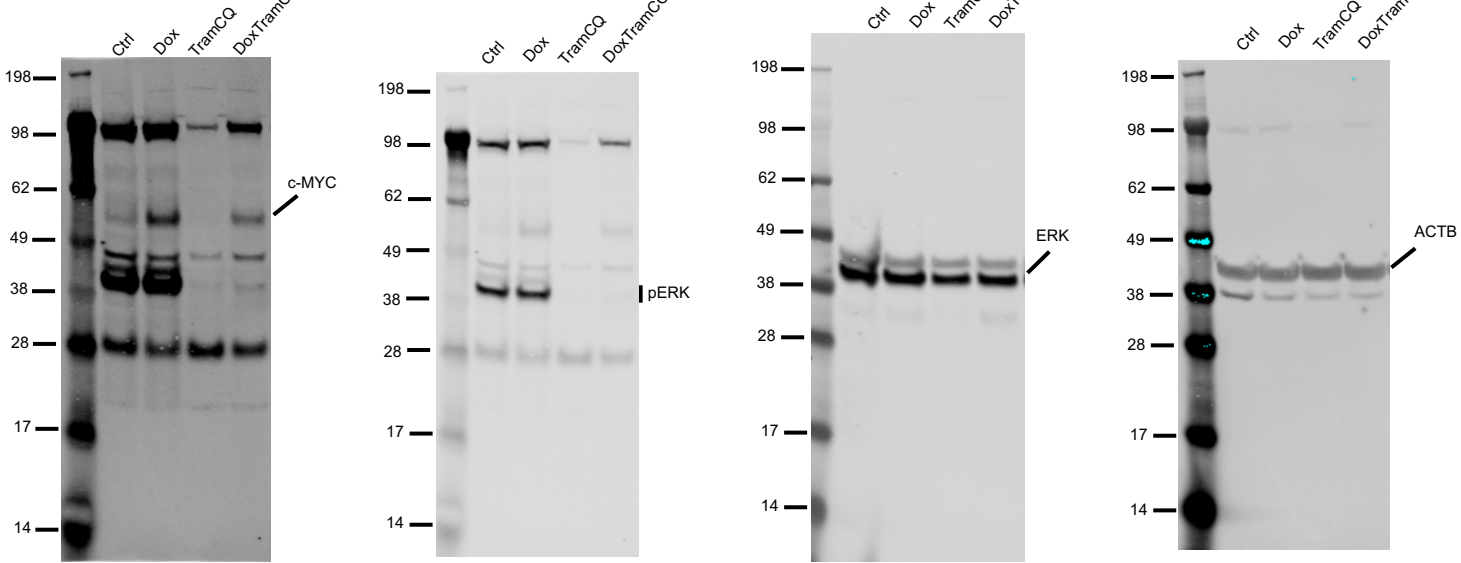

F2E

PANC-1

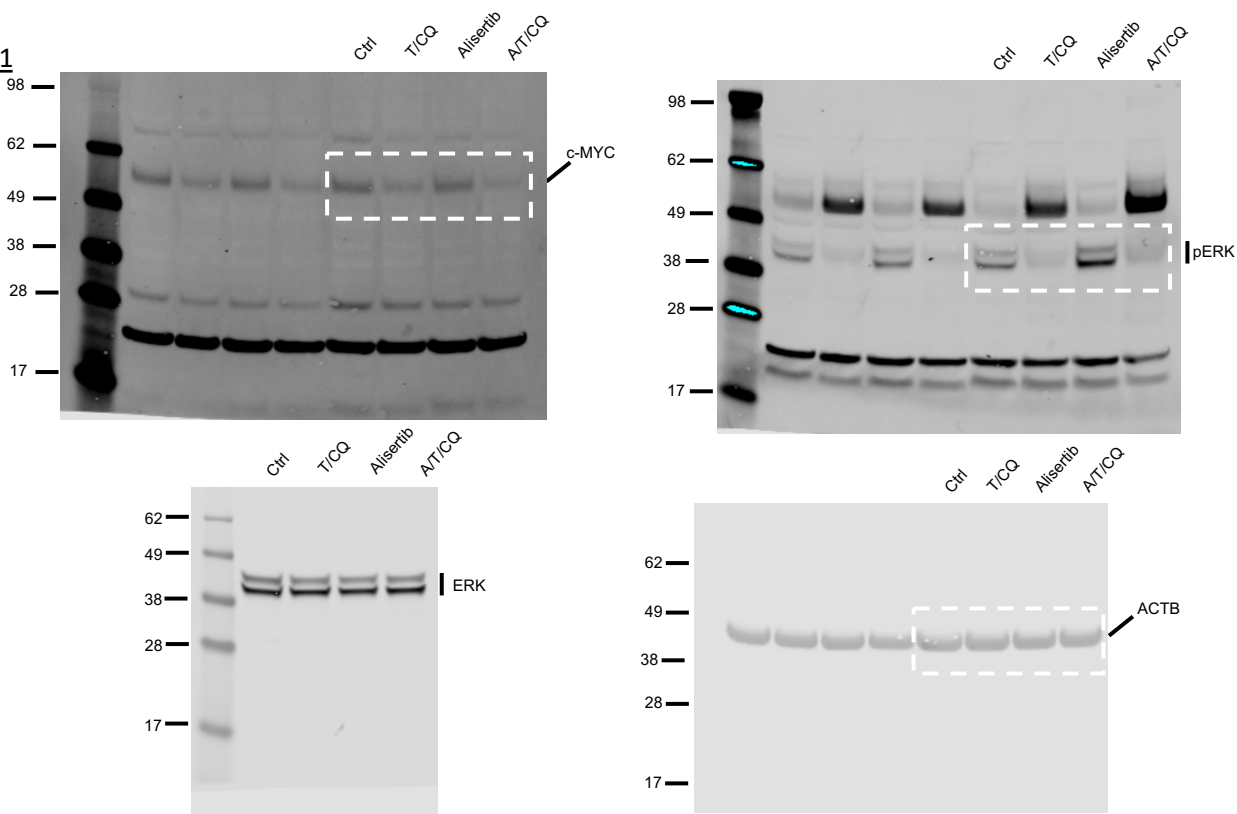

Supplement: SourceData F2 — contains original blots for Fig. 2. [file JEM_20221524_SourceDataF2.pdf]

F3C Panc10.05 Tet-On c-MYC

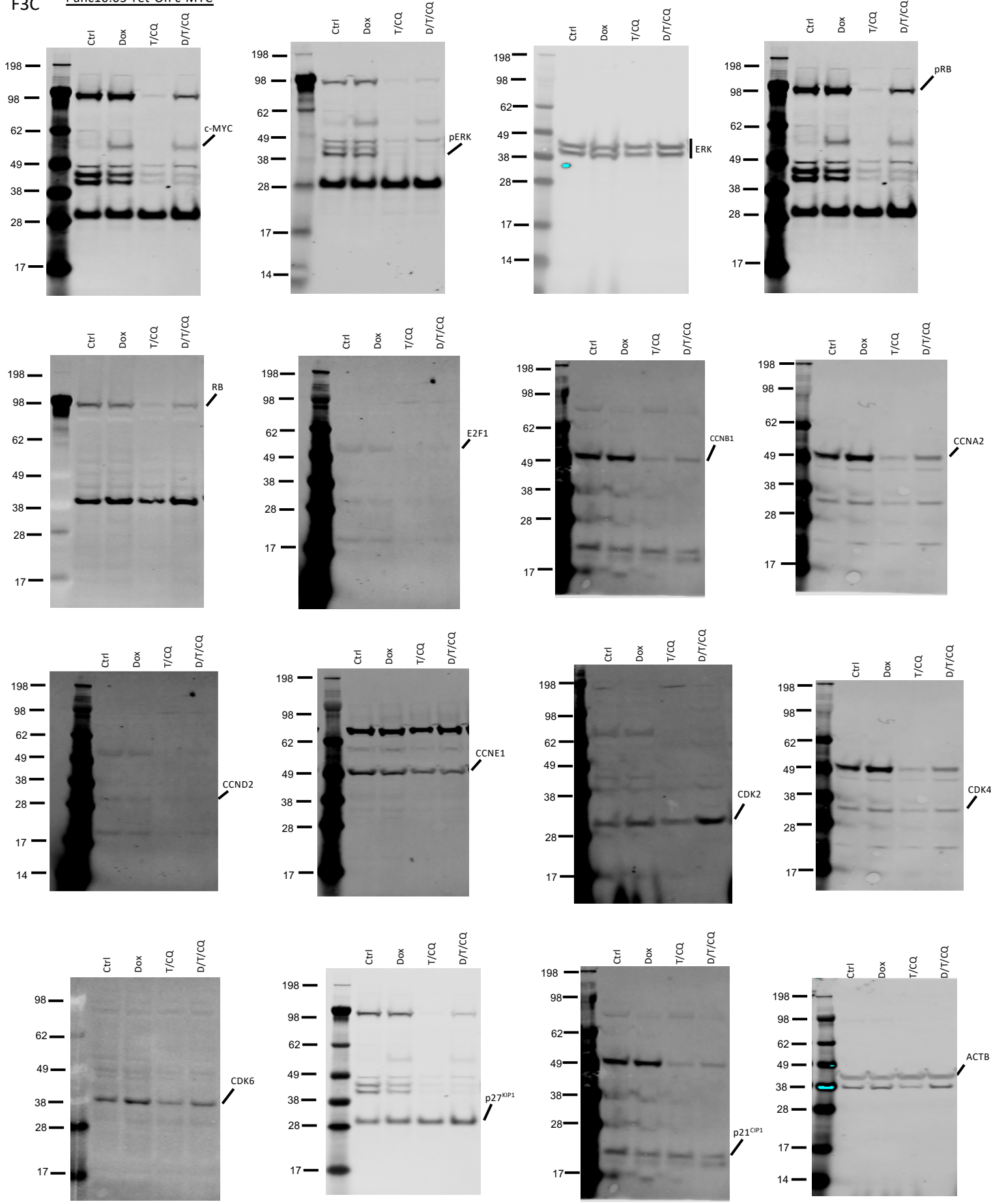

F3C HPAF-II Tet-On c-MYC

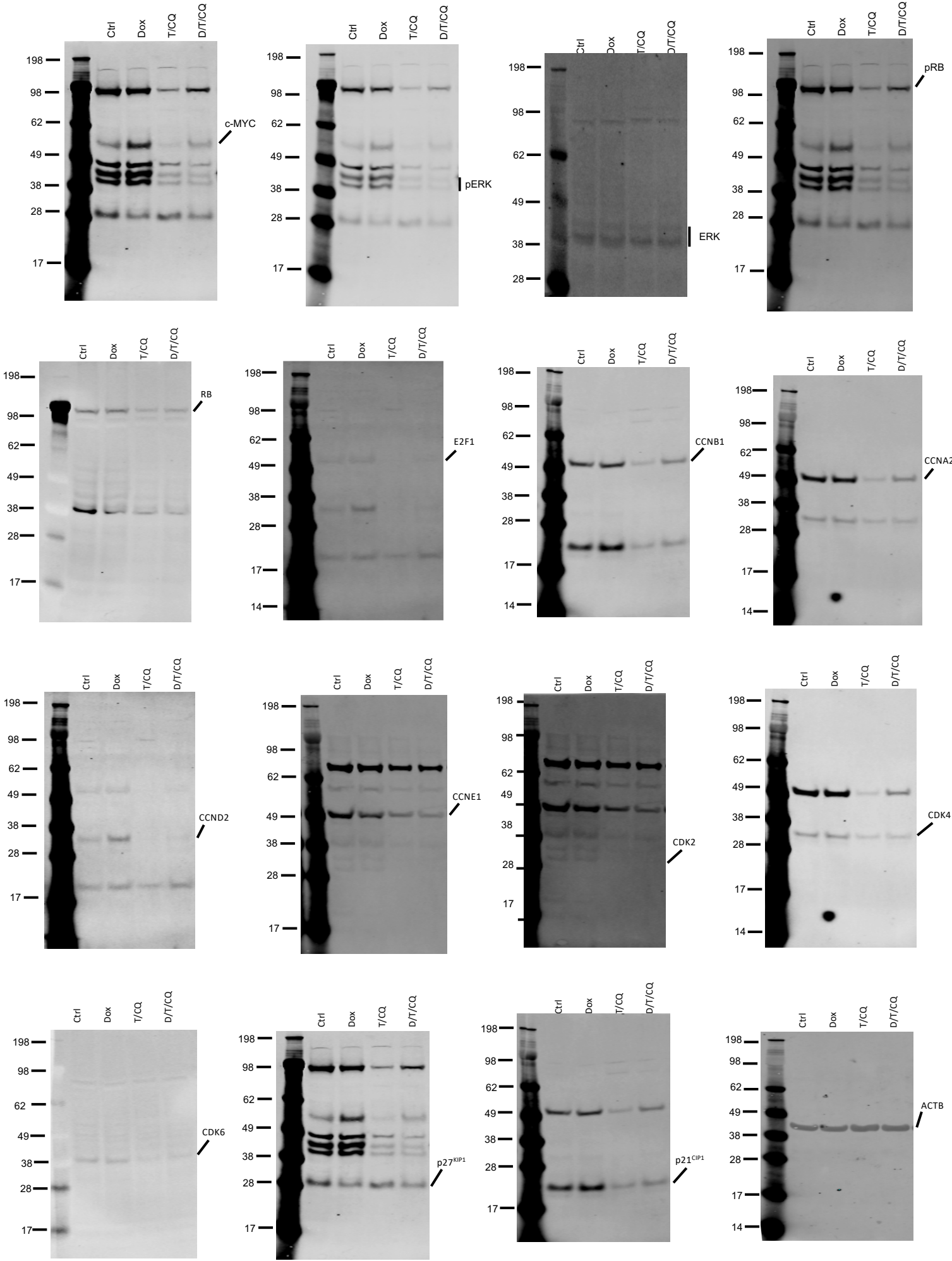

F3C

PDX220 Tet-On c-MYC

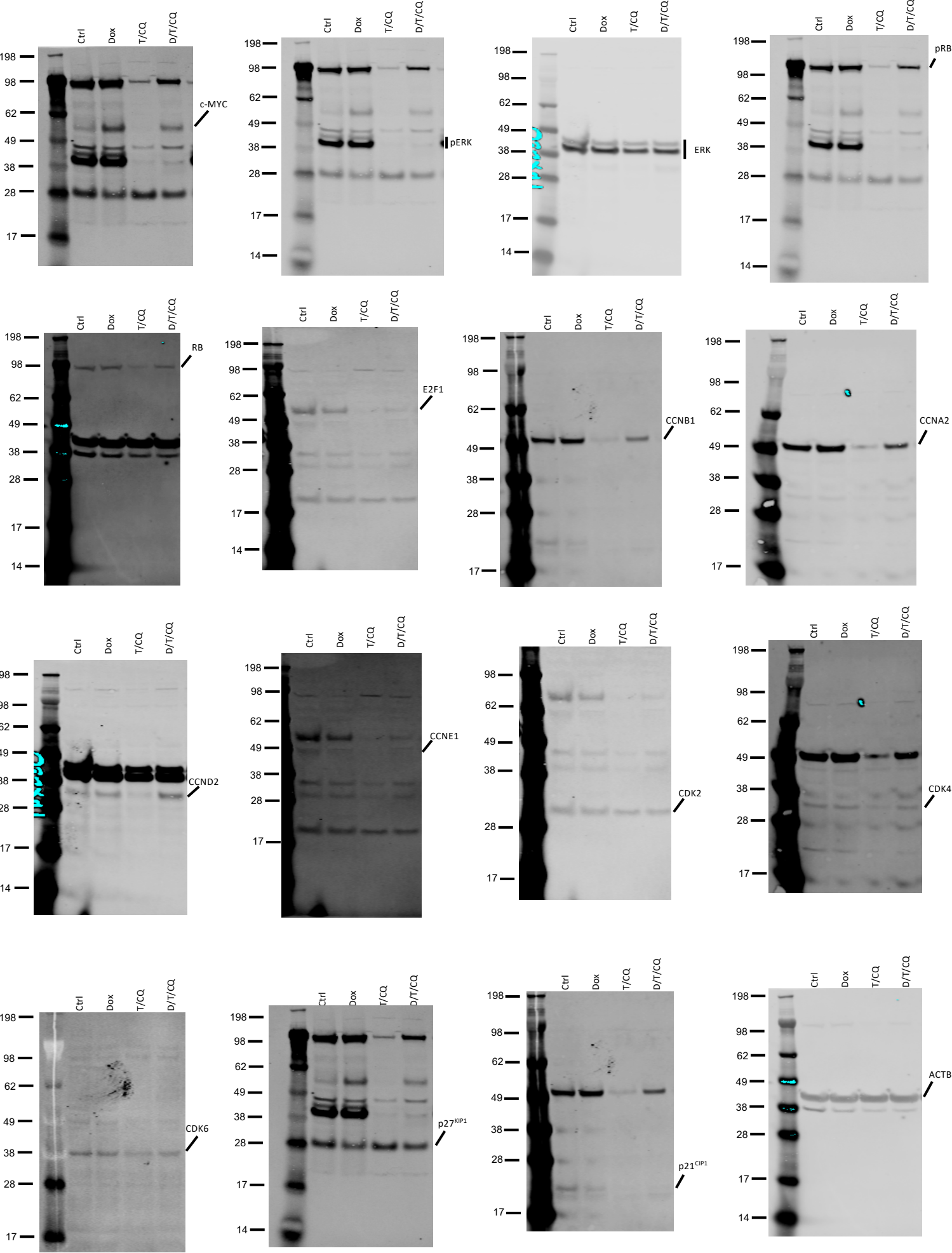

Supplement: SourceData F3 — contains original blots for Fig. 3. [file JEM_20221524_SourceDataF3.pdf]

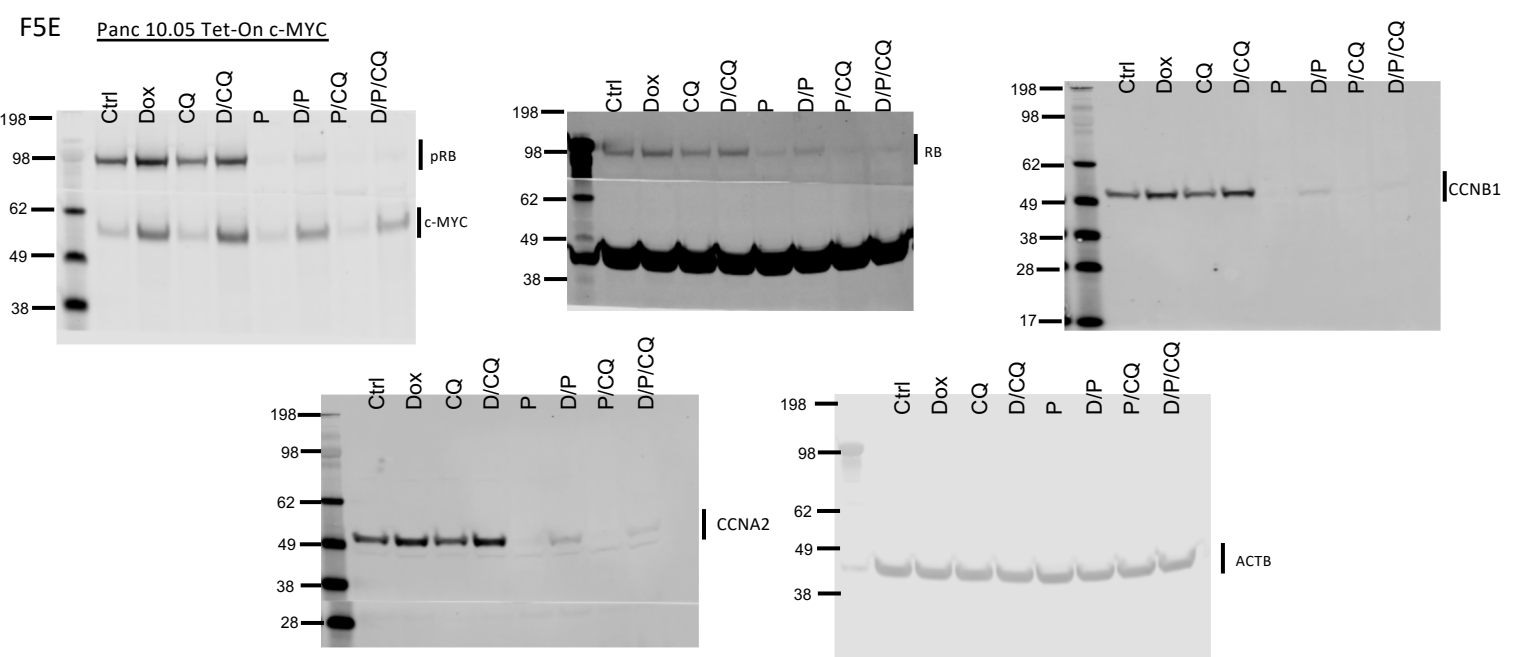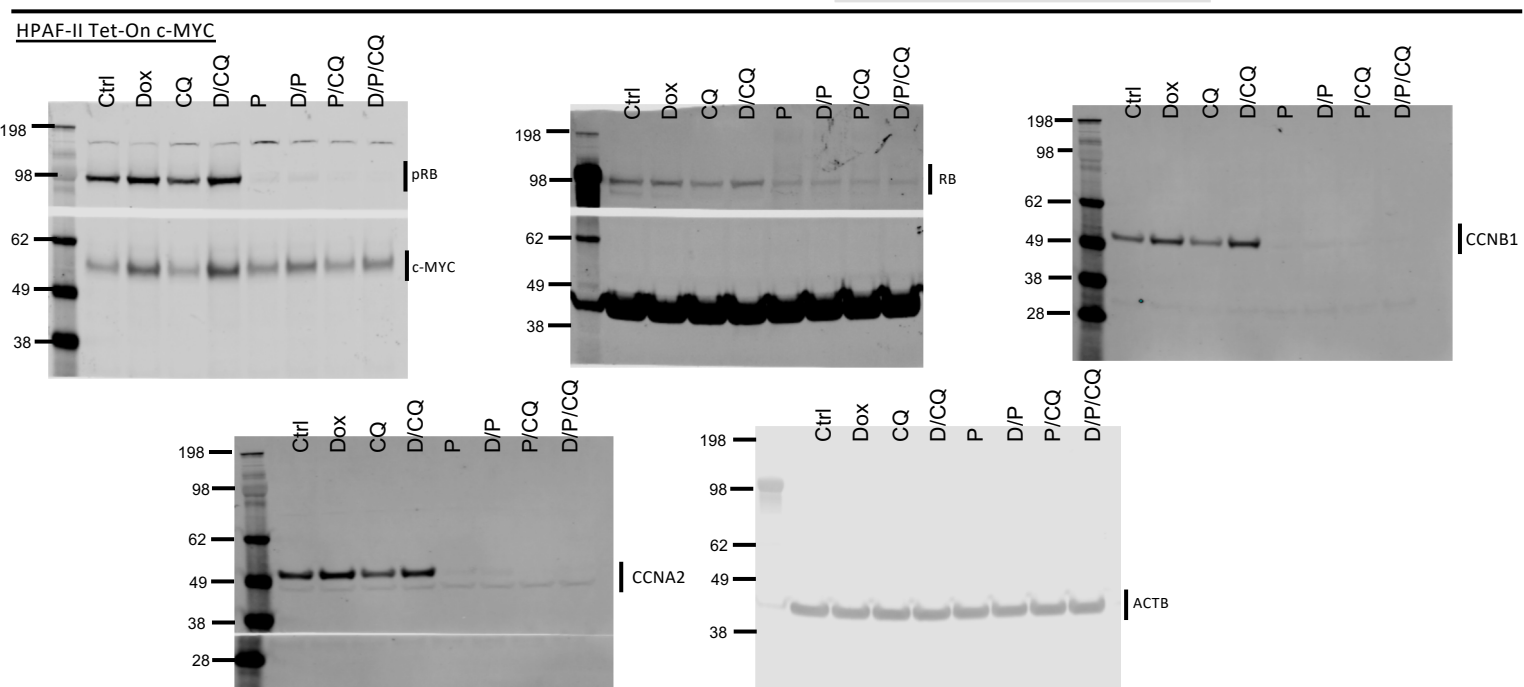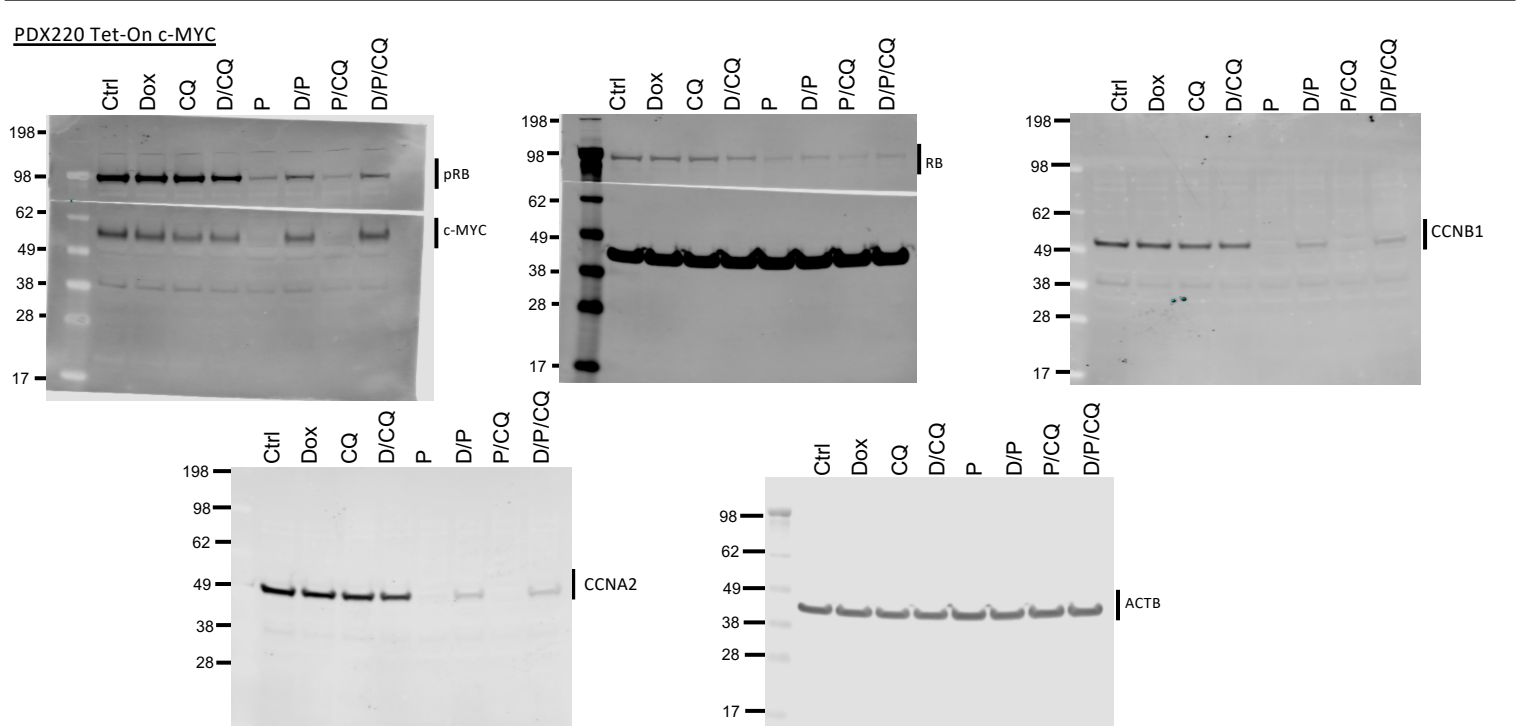

Supplement: SourceData F5 — contains original blots for Fig. 5. [file JEM_20221524_SourceDataF5.pdf]

HPAF-II

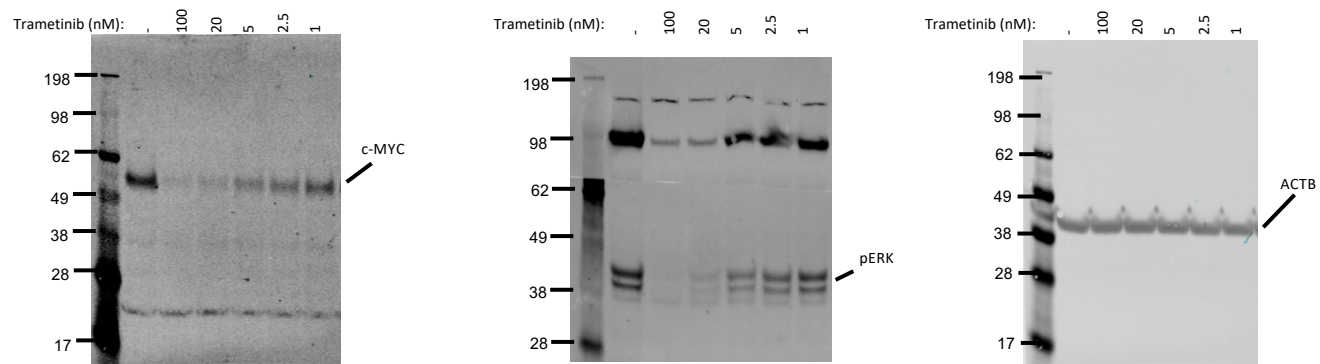

Panc 10.05

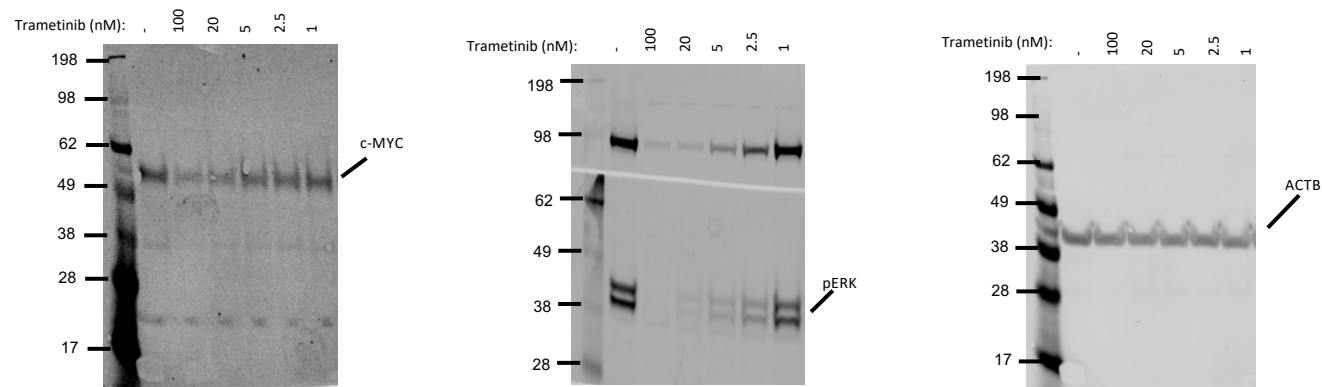

PDX220

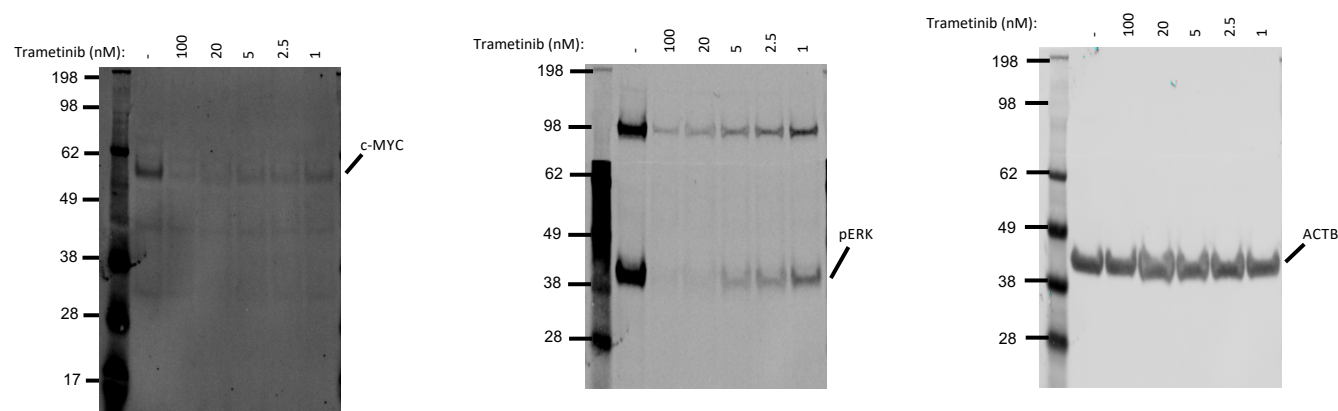

FS1B

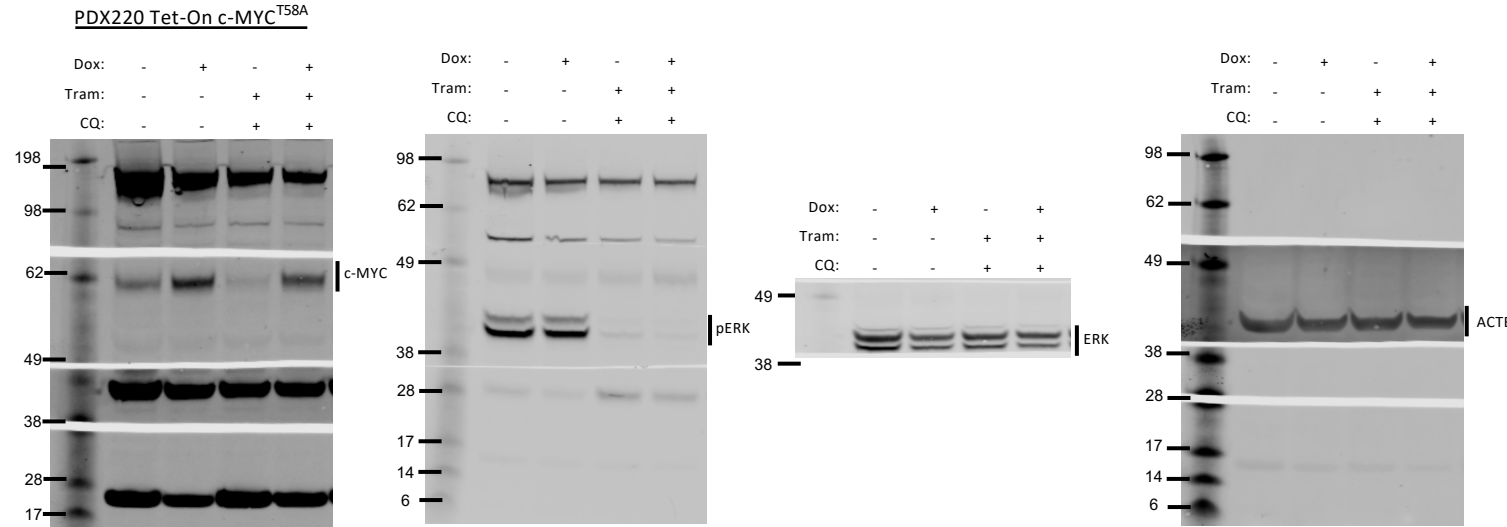

FS1I

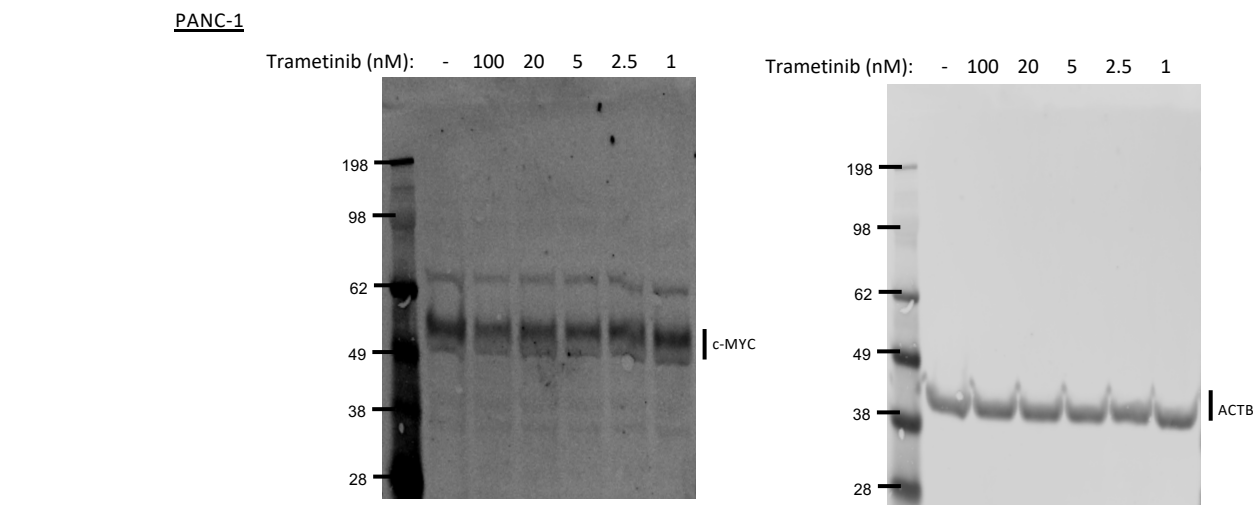

FS1J

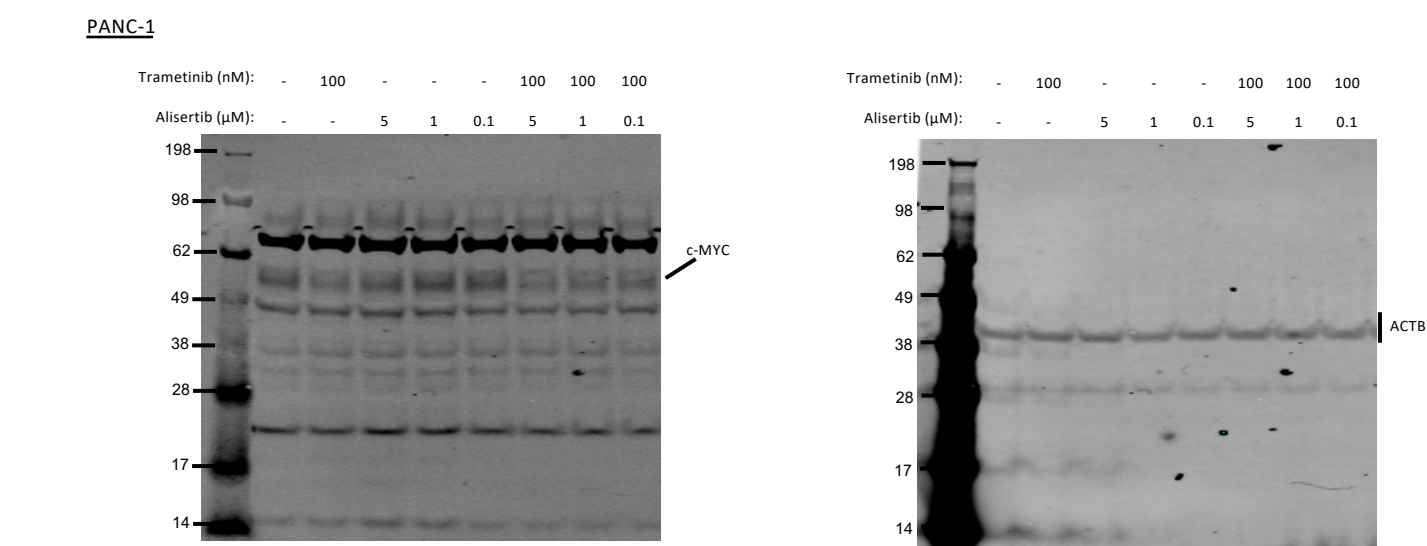

Supplement: SourceData FS1 — contains original blots for Fig. S1. [file JEM_20221524_SourceDataFS1.pdf]
