# Supplementary material for: MYC-mediated resistance to trametinib and HCQ in PDAC is overcome by CDK4/6 and lysosomal inhibition
Source: J Exp Med. 2023 Jan 31;220(3):e20221524. doi: 10.1084/jem.20221524 (PMC9930170; doi:10.1084/jem.20221524)

FS2H

Panc10.05 Tet-On c-MYC^{T58A}

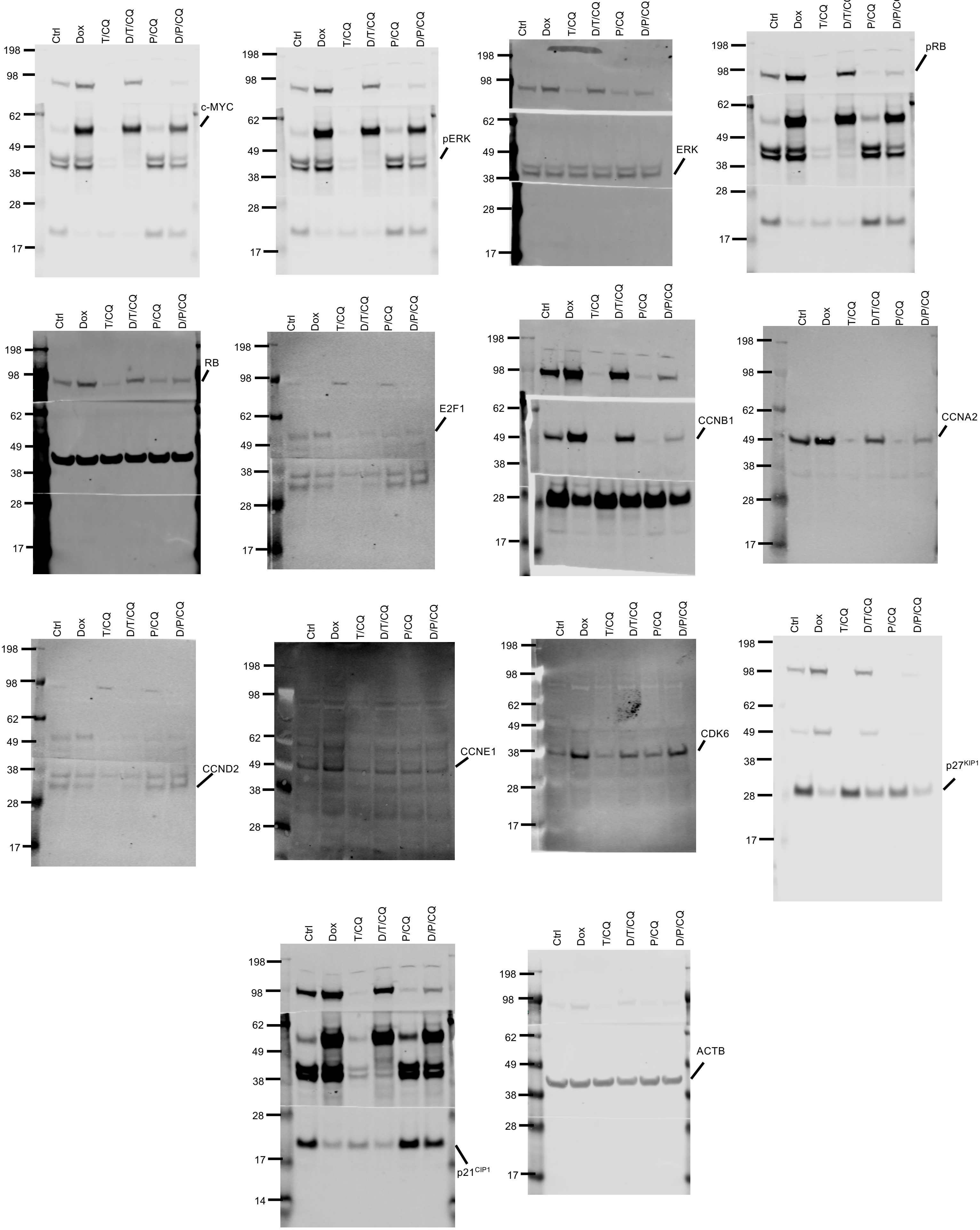

FS2I

HPAF-II Tet-On c-MYC^{T58A}

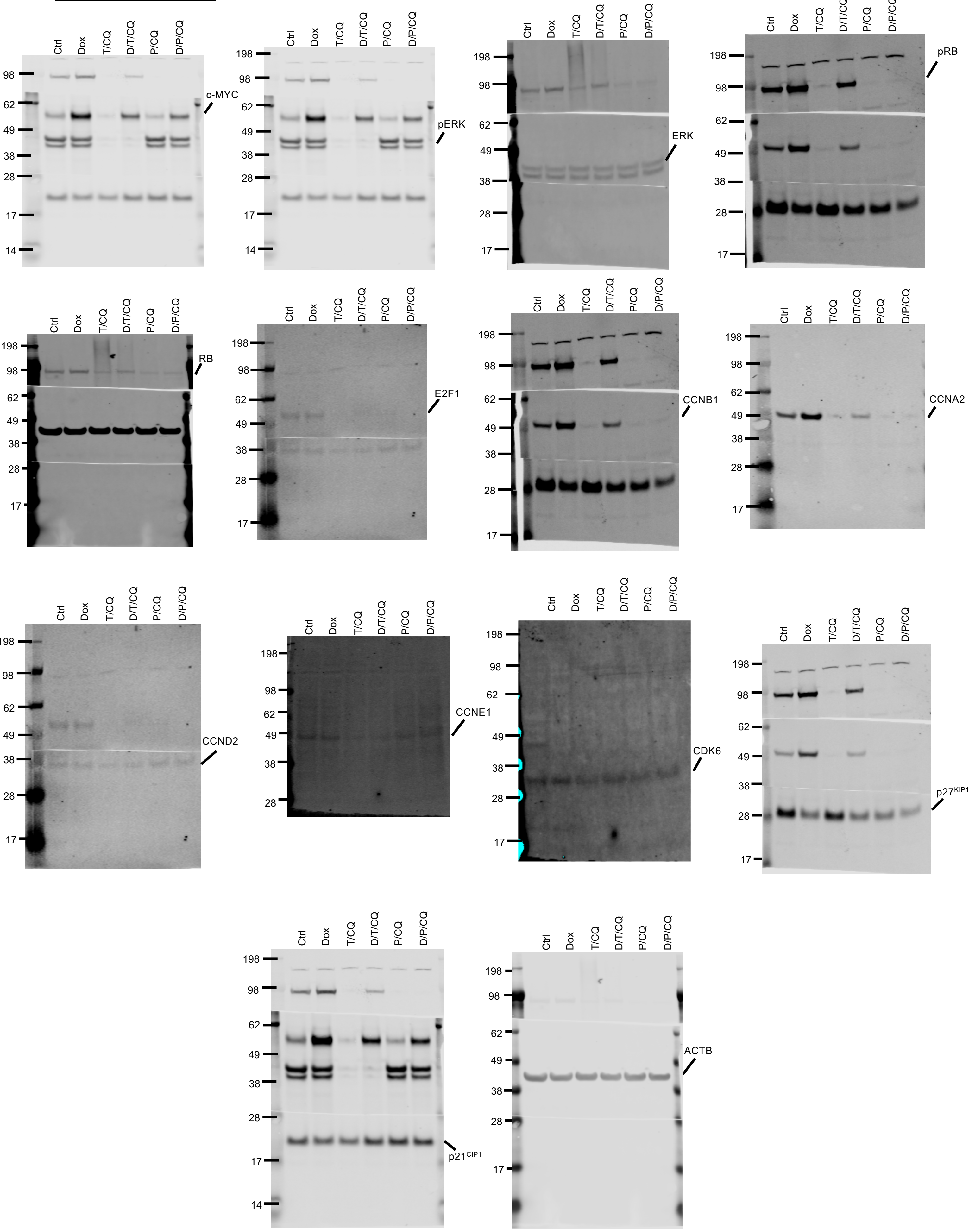

FS2J

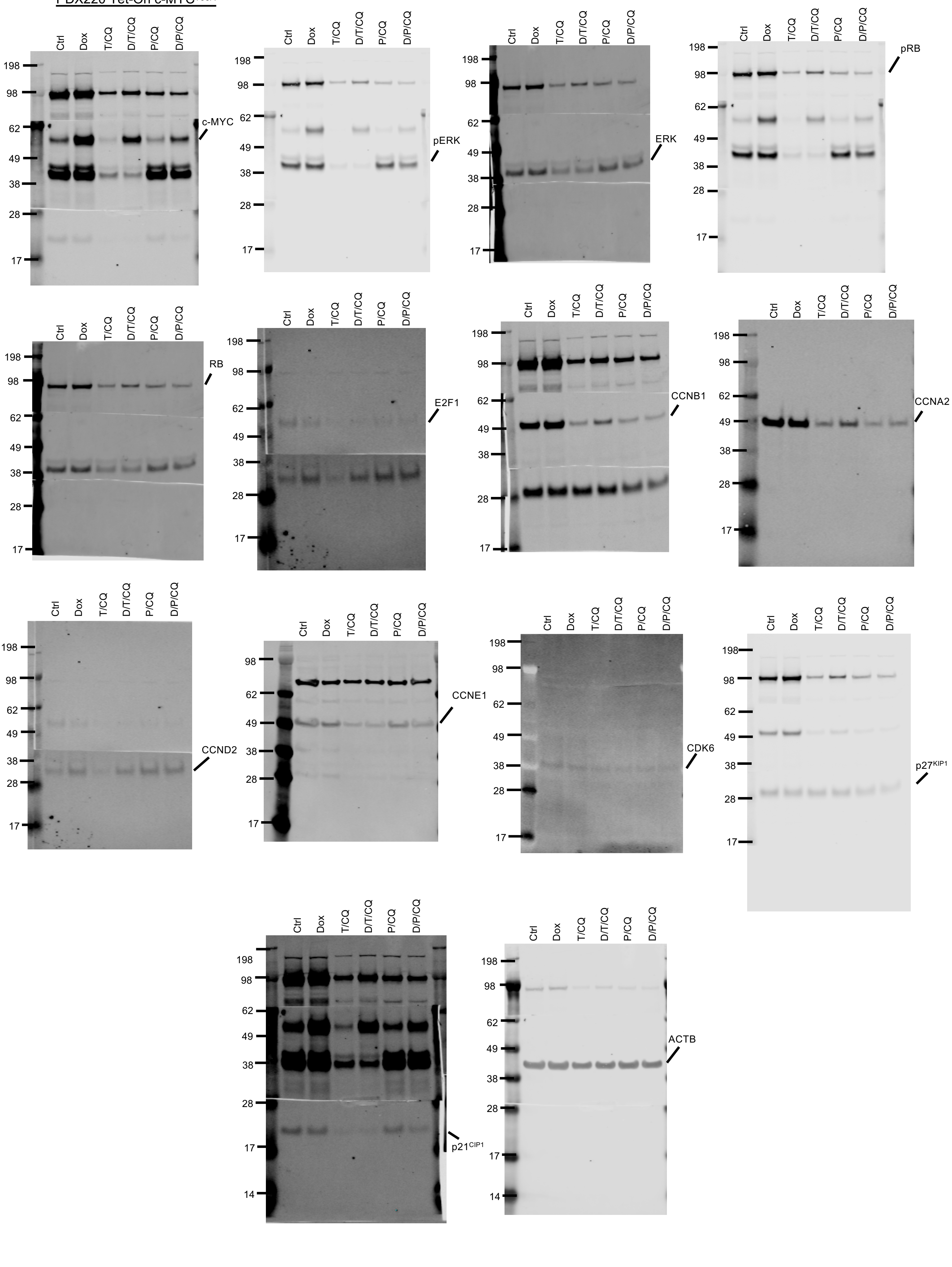

PDX220 Tet-On c-MYC^{T58A}
Ctrl
Dox
T/CQ
D/T/CQ
P/CQ
D/P/CQ
198
98
62
49
38
28
17
14
c-MYC
pERK
ERK
pRB
RB
E2F1
CCNB1
CCNA2
CCND2
CCNE1
CDK6
p27^{KIP1}
p21^{CIP1}
ACTB

FS2K

HPAF-II Tet-On c-MYC

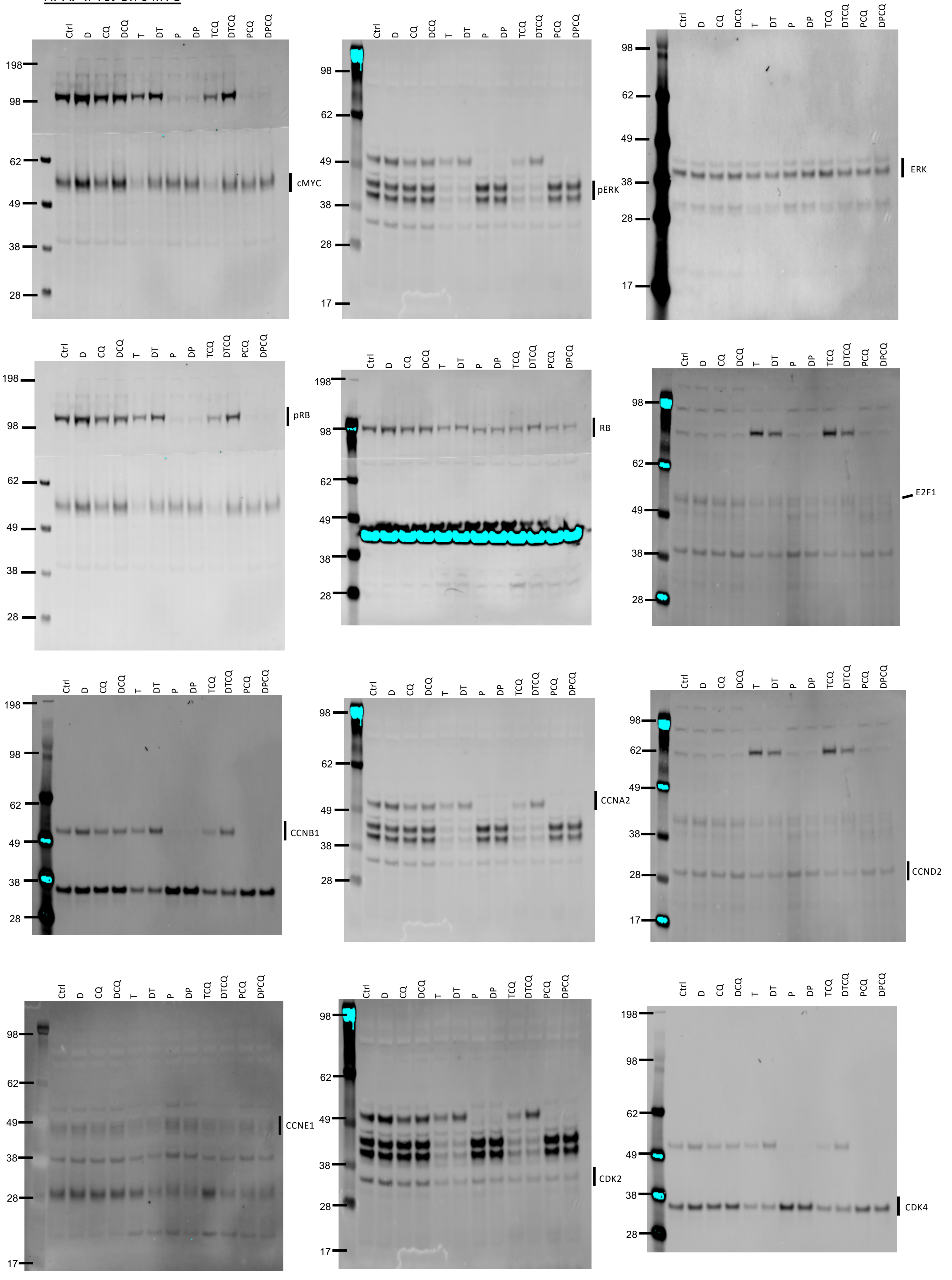

FS2K

HPAF-II Tet-On c-MYC

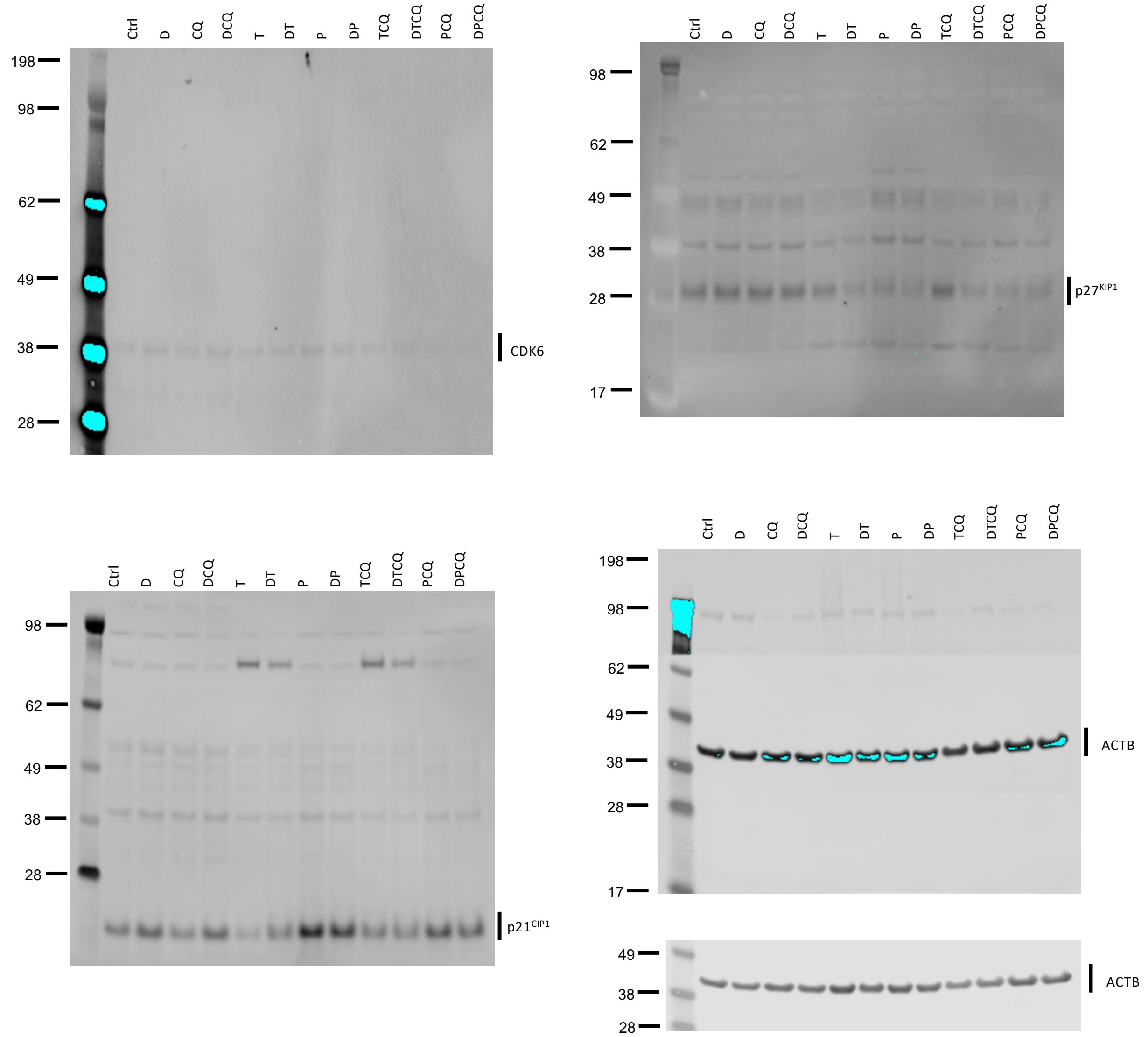

FS2L

PDX220 Tet-On c-MYC

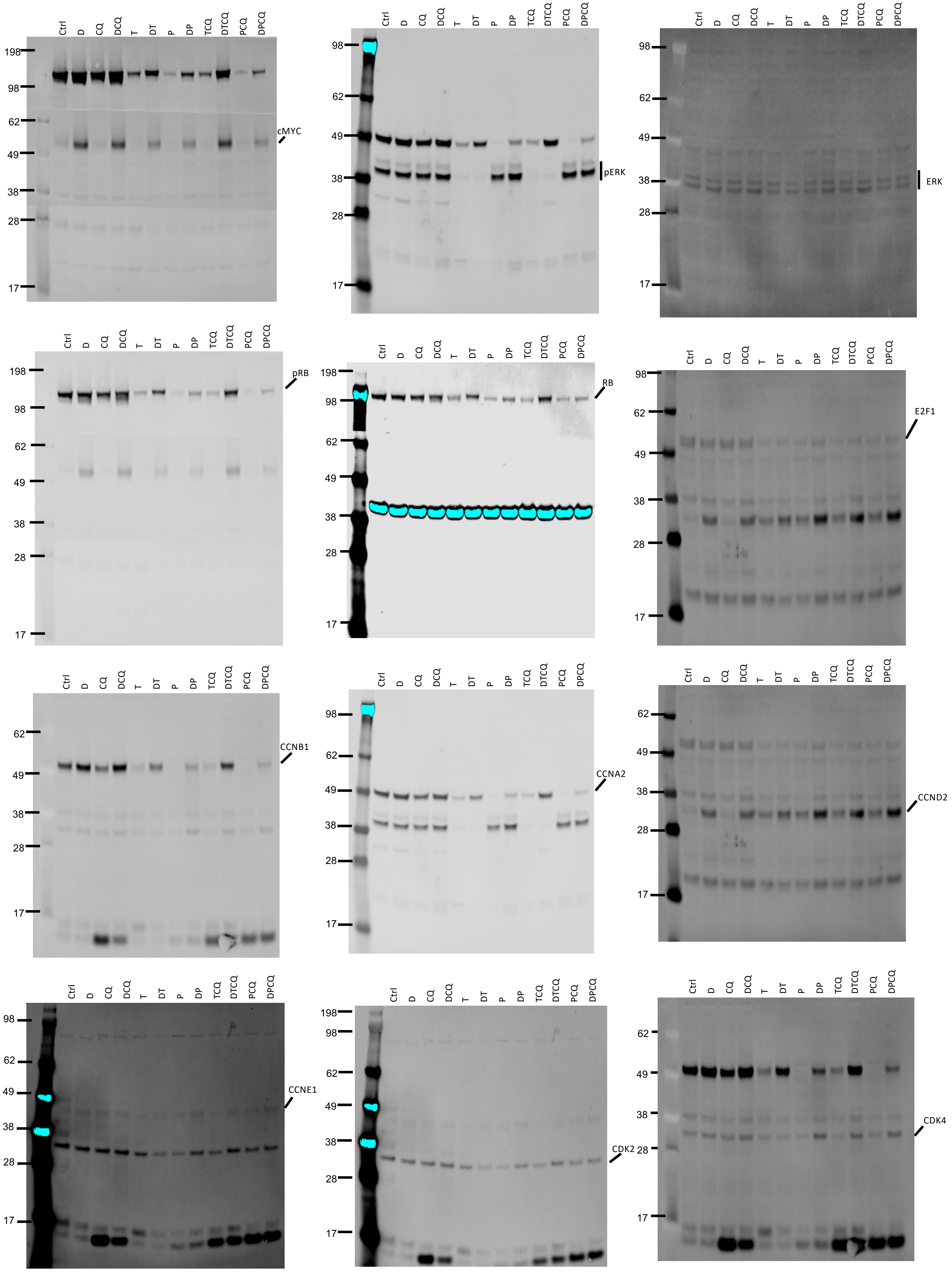

FS2L

PDX220 Tet-On c-MYC

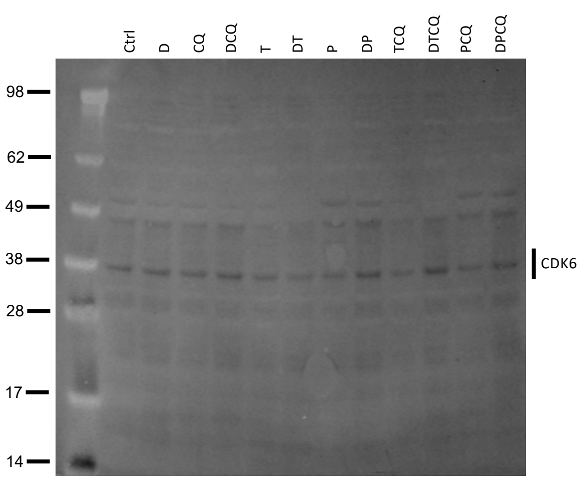

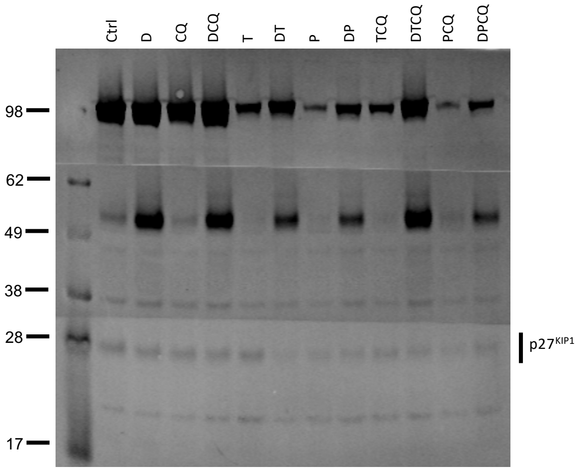

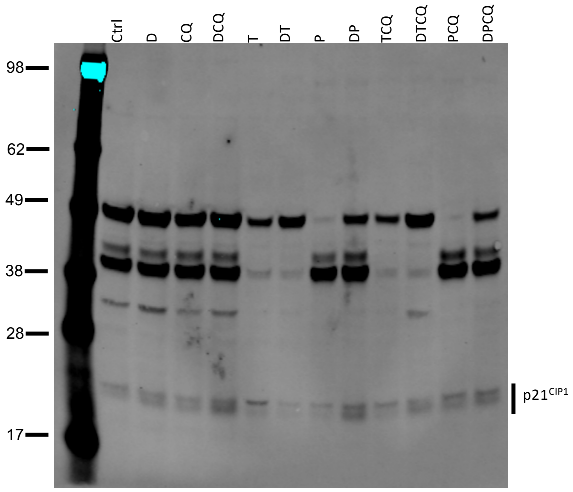

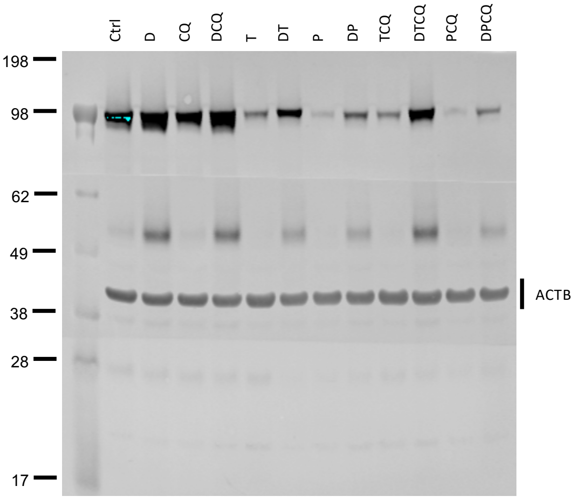

Supplement: SourceData FS2 — contains original blots for Fig. S2. [file JEM_20221524_SourceDataFS2.pdf]
